# Effect of structural variation in the promoter region of *RsMYB1.1* on the skin color of radish taproot

**DOI:** 10.3389/fpls.2023.1327009

**Published:** 2024-01-08

**Authors:** Jiin Kim, Hoyeol Jang, Sun Mi Huh, Ara Cho, Bomi Yim, Seung-Hoon Jeong, Haneul Kim, Hee-Ju Yu, Jeong-Hwan Mun

**Affiliations:** ^1^ Department of Life Sciences, Institute of Convergence Science & Technology, The Catholic University of Korea, Bucheon, Republic of Korea; ^2^ Department of Bioscience and Bioinformatics, Myongji University, Yongin, Republic of Korea; ^3^ Division of Forest Biodiversity, Korea National Arboretum, Pocheon, Republic of Korea; ^4^ Department of Medical and Biological Sciences, The Catholic University of Korea, Bucheon, Republic of Korea

**Keywords:** radish, red skin color, anthocyanin, GWAS, presence/absence variation, *RsMYB1.1*, promoter

## Abstract

Accumulation of anthocyanins in the taproot of radish is an agronomic trait beneficial for human health. Several genetic loci are related to a red skin or flesh color of radish, however, the functional divergence of candidate genes between non-red and red radishes has not been investigated. Here, we report that a novel genetic locus on the R2 chromosome, where *RsMYB1*.*1* is located, is associated with the red color of the skin of radish taproot. A genome-wide association study (GWAS) of 66 non-red-skinned (nR) and 34 red-skinned (R) radish accessions identified three nonsynonymous single nucleotide polymorphisms (SNPs) in the third exon of *RsMYB1*.*1*. Although the genotypes of SNP loci differed between the nR and R radishes, no functional difference in the RsMYB1.1 proteins of nR and R radishes in their physical interaction with RsTT8 was detected by yeast-two hybrid assay or in anthocyanin accumulation in tobacco and radish leaves coexpressing RsMYB1.1 and RsTT8. By contrast, insertion- or deletion-based GWAS revealed that one large AT-rich low-complexity sequence of 1.3–2 kb was inserted in the promoter region of *RsMYB1*.*1* in the nR radishes (*RsMYB1*.*1^nR^*), whereas the R radishes had no such insertion; this represents a presence/absence variation (PAV). This insertion sequence (RsIS) was radish specific and distributed among the nine chromosomes of *Raphanus* genomes. Despite the extremely low transcription level of *RsMYB1*.*1^nR^* in the nR radishes, the inactive *RsMYB1*.*1^nR^* promoter could be functionally restored by deletion of the RsIS. The results of a transient expression assay using radish root sections suggested that the RsIS negatively regulates the expression of *RsMYB1*.*1^nR^*, resulting in the downregulation of anthocyanin biosynthesis genes, including *RsCHS*, *RsDFR*, and *RsANS*, in the nR radishes. This work provides the first evidence of the involvement of PAV in an agronomic trait of radish.

## 1 Introduction

Understanding the genetic bases of agronomically important traits and mining of related genes is a focus of crop science and agriculture. Genomic studies of crop species have focused on the construction of genome assemblies and systemic analyses of genetic resources. The availability of high-quality reference genomes and cost-effective sequencing and genotyping techniques has not only enabled investigation of genome architecture but has also accelerated population genomics and genome-wide association studies (GWAS) of crop species. The discovery of genetic variations by comparing genomes has enabled the evaluation of genomic differences between genotypes and the identification of trait-related genes. GWASs typically analyze single nucleotide polymorphisms (SNPs), however, SNPs do not capture all of the genomic variation that results in phenotypic differences (Saxena et al., 2014; Alseekh et al., 2021). GWASs based on insertion or deletion (InDel) polymorphisms have identified structural variations (SVs), which contribute to genetic diversity and are determinants of genome structure and function (reviewed in Yuan et al., 2021). SVs are defined as ≥50 bp modifications of DNA between genotypes or individuals. There are several types of SVs including copy number variations (CNVs), presence/absence variations (PAVs), InDels, inversions, and translocations. SVs can change the effects of mutations in multiple parts of cis-regulatory regions on gene expression (Alonge et al., 2020) or lead to phenotypic variation in a species as a result of gene loss, gene duplication, the generation of novel genes, and complex rearrangements (Cook et al., 2020). In several plant species, SVs regulate agronomic traits, such as fruit shape in tomato (Xiao et al., 2008) and peach (Guan et al., 2021), fruit texture in apple (Wu et al., 2023), fruit color in apple (Espley et al., 2009) and peach (Guo et al., 2020), leaf shape in rapeseed (Hu et al., 2018), and resistance to biotic stress in maize (Zuo et al., 2015), sorghum (Gobena et al., 2017), and rice (Ashikawa et al., 2008).

*Raphanus sativus* (radish, 2n = 18) belongs to the genus *Raphanus* in the Brassicaceae family. It is closely related to the diploid *Brassica* species (*B. rapa*, *B. nigra*, and *B. oleracea*) and originated from the *Brassica* B genome lineage (Cho et al., 2022). Divergence of *R*. *sativus* from the wild species (*R*. *raphanistrum*) likely occurred during the Quaternary glaciation beginning 2.4 million years ago (Mya), presumably as a result of climate change (Zhang et al., 2021). *R. sativus* is an ancient domesticated species native to both the Mediterranean region and Eastern Asia (Becker, 1962; George and Evans, 1981; Kaneko and Matsuzawa, 1993). Targeted breeding linked with domestication has led to the development of a variety of radish lines and cultivars. *R*. *sativus* is a commercially important root vegetable crop that is cultivated globally, accounting for 2% of the total global production of vegetables; the fresh-radish market was worth 1264 million USD in 2021 (Kopta and Pokluda, 2013; Verified Market Research, 2023) Genomics tools and plant collections are essential for radish research and breeding. Genome sequencing has yielded at least 16 genome assemblies of *Raphanus* species. A comparison of genome assemblies revealed that the genome size of radish ranged from 430–515 Mb with approximately 42,000–53,000 predicted genes, the numbers of which did not deviate from the average by >6% (Zhang et al., 2021; Cho et al., 2022), suggesting genomic variation between genotypes. A *Raphanus* pan-genome study using 11 genome assemblies reported an average of 5.6–10.1 SNPs and 1.2–2.3 InDels per kb between each pair of genomes and 7–11% of genes were potentially affected by SVs. The genes affected by variation were enriched in disease-resistance genes and gibberellin-related genes, suggesting that genetic variation contributes to phenotypic divergence and the adaptive evolution of radish. In parallel with genome sequencing and genotyping tools, conservation of genetic resources is ongoing. The collections reported to date include the Radish Core Collection of 125 accessions from South Korea (Lee et al., 2018; Huh et al., 2023), the National Plant Germplasm System collection of 152 accessions from the United States (Arro and Labate, 2022), and the Core Germplasm Collection comprising 217 accessions from China (Li et al., 2023).

Anthocyanins are natural antioxidant phenolic pigments found in many plant species. In the red radish taproot, anthocyanins accumulate in the vacuoles of skin or flesh cells. The radish anthocyanins are raphanusins, i.e., glucosylated pelagonidin anthocyanins acylated with 4-coumaric acid (Ishikura and Hayashi, 1963; Giusti et al., 1999). Anthocyanins are produced in a branch of the phenylpropanoid pathway, which is involved in the biosynthesis of several flavonoids. One molecule of 4-coumaroyl-CoA is condensed with three molecules of malonyl-CoA by chalcone synthase (CHS) to form naringenin chalcone, which is converted into naringenin by chalcone isomerase. Naringenin is converted into dihydroflavonols by hydroxylation via flavonoid 3’-hydroxylase and flavonoid 3,’ 5’-hydroxylase. Dihydroflavonols are reduced by dihydroflavonol 4-reductase (DFR) to form leucoanthocyanidins. Oxidation of leucoanthocyanidins by anthocyanidin synthase (ANS) generates anthocyanidins, which are highly unstable. Flavonoid glucosyltransferase rapidly catalyzes glycosylation of C3 in ring C or other positions of anthocyanidins to form anthocyanins. O-Methyltransferase and anthocyanin acyltransferase methylate and acylate, respectively, the hydroxyl groups of rings A and B (reviewed by Zha and Koffas, 2017). The expression of structural genes of the phenylpropanoid pathway is coordinated and regulated transcriptionally by a ternary transcription factor (TF) complex named the MBW complex, which is made up of R2R3-MYB TF, basic helix-loop-helix (bHLH) TF, and tryptophan-aspartic acid repeat protein WDR or WD40 (Hichri et al., 2011). The MBW complex interacts with the MYB-recognition element (ANCNNCC) and the bHLH-recognition element (CACN[A/C/T][G/T]) in the promoter regions of the structural genes of the anthocyanin biosynthesis pathway (Zhu et al., 2015). In radish, RsMYB1 promotes anthocyanin biosynthesis by interacting with RsTT8 (Kim et al., 2021). At least three RsMYB1, namely RsMYB1.1 (Liu et al., 2019a), RsMYB1.3 (Tao et al., 2022), and RsMYB1.4 (Yi et al., 2018; Kim et al., 2021), have been identified by genetic mapping, molecular genetics, and SNP GWAS of red radish. Although RsMYB1.3 and RsMYB1.4 of red radish reportedly interact with RsTT8 to modulate anthocyanin biosynthesis in radish taproot (Kim et al., 2021; Tao et al., 2022), whether their counterpart genes in non-red radish are functional is unknown.

According to the annotations of gene models in the Rs2.0 radish genome assembly of cv. WK10039 (Cho et al., 2022), the genes R2.009390 (Rs094840 of Rs1.0) and R7.017240 (Rs388430 of Rs1.0) encode RsMYB1.1 and RsMYB1.4, respectively. These are candidate genes related to a red skin or flesh color, however, their functions in WK10039, which has a taproot with white skin and flesh, are unknown. We performed SNP and InDel GWASs of 100 accessions, which identified a genetic locus associated with the red skin color of radish taproot. This locus encompasses *RsMYB1*.*1*, which has sequence variation between non-red-skinned (nR) and red-skinned (R) radishes. There was no functional difference between the RsMYB1.1 proteins of nR and R radishes. By contrast, a PAV in the promoter region of *RsMYB1*.*1* in nR radish negatively regulated the transcription of the coding sequence (CDS), thus suppressing anthocyanin biosynthesis. This is the first report that the PAV in the promoter region of *RsMYB1.1* rather than the presence of specific type of *RsMYB1*.*1* is responsible for the red color of the skin of radish taproot.

## 2 Materials and methods

### 2.1 Plant materials and nucleic acid extraction

Seeds of each radish inbred line and tobacco (*Nicotiana benthamiana* and *N*. *tabacum*) were germinated on horticultural potting soil (Nongwoo Bio, Suwon, South Korea) in a growth chamber set at 22°C under a 16 h light/8 h dark cycle and 60% humidity. Radish seedlings were transferred to an open field 1 week after germination and grown for 1 to 2 months. Tobacco seedlings were grown in a growth chamber under the same conditions. Genomic DNA (gDNA) was extracted from the leaves of 1-month-old plants using the DNeasy Plant Maxi Kit (Qiagen, Germantown, MD, USA). Total RNA was extracted from plant tissues using TRIzol reagent (Thermo Fisher Scientific, Waltham, MA, USA) and treated with DNase using the TURBO DNA-Free Kit (Thermo Fisher Scientific). The quality and quantity of extracted nucleic acids were analyzed using the Agilent 2100 Bioanalyzer (Agilent, Santa Clara, CA, USA).

### 2.2 Genome-wide association study

The skin color of the taproot of radish accessions was examined 50 to 70 days after planting in the field. For genotype data, we used previously generated whole-genome resequencing data (Kim et al., 2016; Huh et al., 2023). Briefly, approximately 23-fold the average genome coverage (the estimated *R. sativus* genome size is 510.8 Mb) of the quality filtered paired-end (PE) Illumina HiSeq reads from 100 accessions were mapped to the Rs2.0 reference genome (Cho et al., 2022) using BWA-MEM 0.7.12 (Li, 2013) with the parameters of k13, c1000, r10, v1, mapping quality 40, and 99.99% probability. Duplicate alignments were removed using the Picard v2.2.4 package (http://broadinstitute.github.io/picard). For SNP calling, the GATK v3.7.0 toolkit (Mckenna et al., 2010) was used. The HaplotypeCaller and GenotypeGVCFs modules were used to call variant sites per sample, generate a per-sample gVCF, and convert variant calls in gVCF into VCF format. The VariantFiltration module with parameters of FS >1.0 and MQ <40 was used to filter variants. VCFtools v0.1.13 (Danecek et al., 2011) with parameters of DP >10 and GQ <20 was used for additional filtering. The SelectVariants module was used to generate a subset VCF file of SNPs. For InDel calling, Pindel v0.2.5b9 (Ye et al., 2009) with parameters of r false, t false, x 4, and w 3 was used. The resulting SNP and InDel variants (1 bp to 3 kb) were further filtered using the variation positions on the chromosomes and PLINK v1.90 software (Purcell et al., 2007) with parameters of mind 0.2 and maf 0.1. GWAS was performed using GEMMA v0.98.5 (Zhou and Stephens, 2012) with the gk 1 parameter for calculating the centered relatedness matrix of SNP and InDel variants with phenotype, respectively, and the lmm 4 parameter for association tests with three univariate linear mixed models (Wald, likelihood ratio, and score tests). The significant and suggestive thresholds values of *P* were set at 0.05 and 1 per the number of SNPs and InDels used in the GWAS (Guo et al., 2017). Manhattan and Quantile-Quantile (Q-Q) graphs based on *P-*values were plotted using the ggplot2 package (http://ggplot2.org).

### 2.3 Genotyping, protein structure prediction, gene cloning, and plasmid construction

The primers used are listed in **Supplementary Table S1**. The target genome regions of each accession were reconstructed by reference-guided assembly. Illumina reads from each accession mapped to the target regions were extracted and assembled into a consensus sequence using Geneious Prime 2023.1.2 (Biomatters, Auckland, New Zealand) with the default parameters. The assembled sequences were multiple-aligned with the reference sequence of WK10039 and genotyping primers were designed based on the aligned sequences using the tools in Geneious Prime. For InDel genotyping, PCR amplification was performed using the gt-pRsMYB1.1 primer pair, which targeted the conserved region of the *RsMYB1*.*1* promoter, followed by cloning and sequencing. For SNP genotyping, multiplex-PCR primer sets including amplification (gt-RsMYB1.1-E3) and extension (e1 to e3) primers were designed to target the third exon of *RsMYB1*.*1* using MassARRAY Typer software (Agena, San Diego, CA, USA). Protein structures were predicted using AlphaFold Protein Structure Database (AlphaFold DB, released on May, 2020) (Jumper et al., 2021) and viewed through YASARA (Land and Humble, 2018). PCR amplification, single-base extension, and genotyping of target loci were performed using the MassARRAY system according to the manufacturer’s instructions (Agena). The CDSs of *RsMYB1*.*1* and *RsTT8* were synthesized and cloned into the pTwist-ENTR vector by Twist Bioscience (South San Francisco, CA, USA). The entry plasmids were recombined with the pK7WG2D vector in the presence of LR Clonase II (Thermo Fisher Scientific) to obtain the binary constructs for overexpression of the *RsMYB1*.*1* and *RsTT8* CDSs under the control of the *CaMV 35S* promoter. To construct the promoter::GUS reporter fusions, the 5’-flanking regions of the *RsMYB1*.*1* genes were amplified from the gDNAs of WK10039 (3.2 kb) and CUR034 (1.9 kb) using Phusion High Fidelity DNA polymerase (Thermo Fisher Scientific) with the c-pMYB1.1^nR^-full and c-pMYB1.1^R^-full primers, respectively. For promoter deletion analysis, the c-pMYB1.1^nR^-d1 to c-pMYB1.1^nR^-d4 primer pairs were used to amplify fragments of the *RsMYB1*.*1* promoter of WK10039. The resulting PCR amplicons were purified by agarose gel electrophoresis, cloned into the All-in-One vector (Biofact, Daejeon, South Korea), and confirmed by sequencing. The cloned fragments were digested with *Eco*RI/*Nco*I and ligated into the pCAMBIA3301 vector. The resulting binary vector constructs were inserted into *Agrobacterium tumefaciens* strain GV3101.

### 2.4 Yeast two-hybrid assay

The CDSs of *RsMYB1*.*1* and *RsTT8* were amplified from the synthetic fragments described above using the y-RsMYB1.1, y-RsTT8^nR^, and y-RsTT8^R^ primer pairs and were inserted in-frame into the prey vector pGADT7 and the bait vector pGBKT7. Pairwise interactions between RsMYB1.1 and RsTT8 were screened in the yeast strains AH109 and PBN204, which were sequentially transformed by the bait vector and the prey vector. Transformed yeasts were cultured on SD Leu– Trp– Ade– or His– medium for AH109 and SD Leu– Trp– Ura– or Ade– medium for PBN204. Colonies positive for PBN204 were confirmed via filter assay using X-gal as the substrate for β-galactosidase.

### 2.5 Transient plant transformation and GUS histochemical analysis

Transgenes were delivered into plant cells using the syringe infiltration method. For leaf infiltration, *A*. *tumefaciens* GV3101 culture with an optical density at 600 nm (OD_600_) of 0.8 in infiltration medium (10 mM MES [pH 5.6], 10 mM MgCl_2_, and 100 mM acetosyringone) was infiltrated into the abaxial side of fully expanded radish and tobacco leaves by pressing the tip of a 1 mL syringe without a needle. For coexpression of two independent vector constructs, an equal volume of *A*. *tumefaciens* suspension with an OD_600_ of 0.8 in infiltration medium was mixed before infiltration. For root-section transformation, the *Agrobacterium* cultures with an OD_600_ of 1.0 were suspended in 0.5× MS medium containing 2% sucrose, 150 mM acetosyringone, and 0.01% Tween 20 (pH 5.8), and infiltrated into 1 mm thick radish taproot sections using a 1 mL syringe without a needle. Typically, multiple injections were performed on a single leaf or taproot section to expand the infiltrated parts. The *Agrobacterium-*infiltrated plant materials were incubated for 3 (GUS analysis) to 5 (anthocyanin quantification) days in a growth chamber and were used to evaluate gene expression. Histochemical and fluorometric analyses of GUS activity were performed as described previously (Alwen et al., 1992) with modifications. For GUS histochemical staining, plant materials were incubated in phosphate buffer (100 mM NaPO_4_ [pH 7.0], 0.5 mM potassium ferricyanide, 0.5 mM potassium ferrocyanide, 10 mM EDTA [pH 8.0], and 0.1% Triton X-100), containing 1 mM 5-bromo-4-chloro-3-indolylglucuronide (X-Gluc; Sigma-Aldrich, Saint Louis, MO, USA) as the substrate, at 37°C for 24 h. Photographs of histochemically stained sections were obtained without fixation using a digital camera (Nikon, Tokyo, Japan). For fluorometric assays, 50 mg plant material ground in liquid nitrogen was incubated in 0.5 mL GUS extraction buffer (50 mM NaH_2_PO_4_ [pH 7.0], 10 mM EDTA, 0.1% Triton X-100, 0.1% sarcosyl, and 10 mM β-mercaptoethanol) containing 4-methylumbelliferyl-β-D-glucuronide (MUG; Sigma-Aldrich) as the substrate at 37°C for 1 h. Fluorescence was measured with excitation at 365 nm and emission at 455 nm using an Infinite M200 PRO Plate Reader (Tecan, Männedorf, Switzerland). GUS values were expressed as nmol 4-methylumbelliferone (4-MU; Sigma-Aldrich) min^–1^ mg^–1^ soluble protein.

### 2.6 Quantification of anthocyanins and quantitative PCR

Radish roots were sampled from 50-day-old plants grown in the field and tobacco leaves were harvested 5 days after *Agrobacterium* infiltration. For quantification of total anthocyanin levels, 0.3 g ground tissue in liquid nitrogen was added to 1 mL 1% (v/v) HCl methanol and incubated for 24 h at 25°C with gentle stirring in the dark. The anthocyanin solution was centrifuged at 4°C and 13,000 × g for 15 min, and the supernatants were subjected to measurement of absorbance at wavelengths of 530, 620, and 650 nm using an SM 1200 UV-Vis Spectrophotometer (AZZOTA Scientific, Claymont, DE, USA). Anthocyanin levels were calculated from three biological replicates using cyanidin-3-glucoside as the reference, according to the following formula (Giusti and Wrolstad, 2001): [(A_530_ − A_620_) − 0.1 × (A_650_ − A_620_)] × MW × DF × 1000 × ∋; where A is supernatant absorbance, wavelengths are indicated as subscripts, MW is the molecular weight of cyanidin-3-glucoside (449.2 g/mol), DF is a dilution factor, and ∋ is the cuvette optical path length (1 cm). Expression values of genes were obtained from previously generated mRNA sequencing (mRNA-seq) data (Jeong et al., 2016). For quantitative PCR (qPCR) analysis, first-strand cDNA was synthesized using total RNA extracted from root tissues with the Revert Aid First-Strand cDNA Synthesis Kit (Thermo Fisher Scientific) according to the manufacturer’s instructions. cDNAs were diluted 10-fold and qPCR was performed using PowerUp SYBR Green Master Mix (Thermo Fisher Scientific), primer pairs designed to amplify target genes, and the StepOne Plus™ Real-Time PCR System (Thermo Fisher Scientific). The 2^-ΔΔCt^ method was used to quantify gene expression with *RsActin7-2* as the reference. To statistically test the difference in gene expression, the independent t-test was performed using Excel.

## 3 Results

### 3.1 *RsMYB1.1* is associated with the taproot skin color of radish

**Figure 1:**
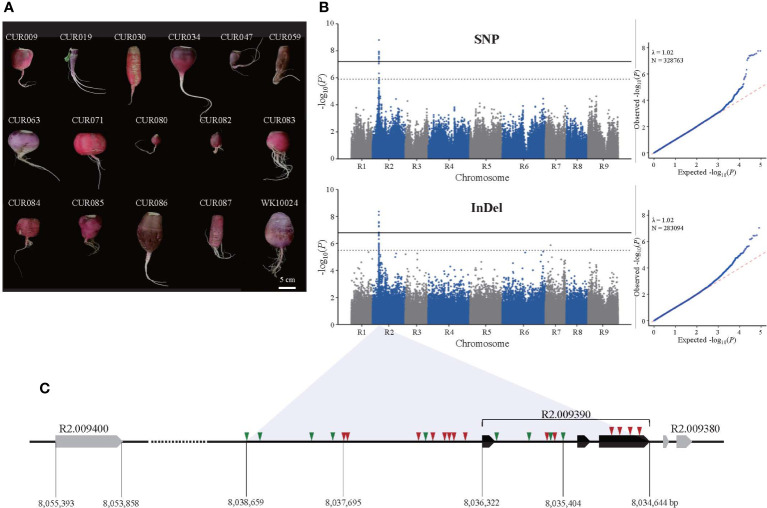
Identification of genomic loci associated with the taproot skin color of radish by GWAS. **(A)** Photographs of selected red-skinned radish accessions in the core collection. **(B)** Manhattan and Q-Q plots of SNP (upper panel) and InDel (lower panel) GWAS. Solid and dotted lines in Manhattan plots depict thresholds for significant and suggestive, respectively. **(C)** Variation in the candidate genome region on the R2 chromosome. Triangles represent SNP (red) and InDel (green) variation.

In total, 100 genotypes, including 83 inbred lines of the core collection (Huh et al., 2023) and 17 previously analyzed genotypes (Kim et al., 2016), were subjected to GWAS of red taproot skin color (**Supplementary Table S2**). In all, 34 genotypes had red skins; the others were white, white-green, or black (**Figure 1A**). The R radishes contained 118.2 mg anthocyanins per 100 g fresh weight (FW) of root on average whereas nR radishes had no anthocyanins (Huh et al., 2023). For genome sequences, we obtained approximately 1170 Gb clean data from 100 genotypes. The average read mapping rate and depth were 98.7% and 26.4-fold, respectively. The coverage per genotype using Rs2.0 as the reference genome was 73.6–98.2%. Most unmapped regions (83.6%) in the Rs2.0 were repetitive, heterochromatic regions. The initial variation calls identified 34,380,875 SNPs by GATK and 1,825,271 InDels by Pindel. After filtering and variant calling, 794,484 SNPs and 308,433 InDels were selected to investigate the association between phenotypes and variation.

The GWAS showed that taproot skin color was associated with a region on the R2 chromosome that spans 7.95 to 8.10 Mb (**Figure 1B**). The SNP-based GWAS identified 127 SNPs associated with the trait, among which 14 SNPs reached the suggestive threshold (–log_10_(*P*) = 5.90). Interestingly, 12 of 14 suggestive SNPs were located in five genes in the genomic region, and the others were in the intergenic region (**Supplementary Table S3**). The lead SNP (–log_10_(*P*) = 8.80) and one significant SNP (–log_10_(*P*) = 7.44) were located in the promoter and the third exon of *R2*.*009390* (MYB1.1). We also annotated suggestive SNPs in the coding regions of *R2*.*009320* (methyltransferase), *R2*.*009350* (bHLH), *R2*.*009400* (DELLA protein RGL1), and *R2*.*009420* (pentatricopeptide repeat-containing protein). The InDel-based GWAS identified 259 associated InDels, 21 of which reached the suggestive threshold (–log_10_(*P*) = 5.49). All of the suggestive InDels were intergenic except two in introns of *R2*.*009320* (2 bp deletion) and *R2*.*009350* (1 bp deletion). In all, 43 InDel loci were identified in the genomic region near *R2*.*009390*, including the promoter and intron (**Supplementary Table S4**). The size differences in these InDels ranged from 1 to 42 bp, indicating high sequence variation. Unfortunately, none reached the suggestive threshold for taproot skin color. Based on the role of MYB1.1 in anthocyanin biosynthesis, we selected *R2*.*009390*, named *RsMYB1*.*1*, and nearby sequences as a candidate region associated with taproot skin color (**Figure 1C**).

### 3.2 Conserved variation patterns in the exon and promoter of *RsMYB1.1*

**Figure 2:**
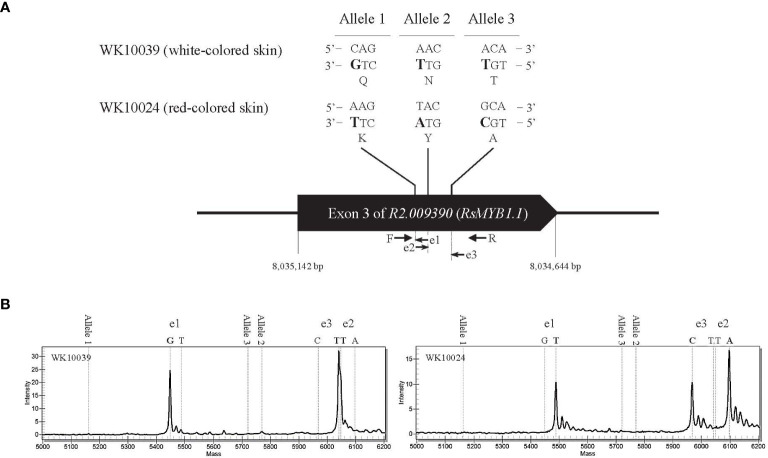
Genotyping of nonsynonymous SNP alleles in the third exon of R2.009390. The target regions of three SNP alleles showing nonsynonymous changes between the nR radish WK10039 and the R radish WK10024 were amplified by PCR and genotyped using the MassARRAY markers. **(A)** Nucleotide and amino acid sequences of SNP alleles. Arrows indicate primers for MassAARY genotyping. **(B)** MassARRAY spectrograms of WK10039 (left) and WK10024 (right). Peaks in the spectrograms represent nucleotides extending from the target alleles.

An in-depth sequence comparison of candidate regions between nR and R radishes was performed using the consensus sequences assembled from the short read sequences of each genotype. In multiple alignment analyses of the corresponding sequences, only *RsMYB1*.*1* and its nearby sequences showed conserved patterns of SNP and InDel variation. In the third exon of *RsMYB1*.*1*, one synonymous SNP locus (Rs:8,034,806 A/G) and three group-specific nonsynonymous SNP loci (allele1, R2:8,034,922 C/A; allele 2, R2:8,034,895 A/T; allele 3, R2:8,034,850 A/G) having different encoded amino acids (allele 1, 158 Q/K; allele 2, 167 N/Y; allele 3, 182 T/A) between the nR and the R radishes were identified (**Figure 2A**). Group-specific SNP types of the loci were conserved in the core collection accessions and the commercial cultivars. Genotyping of the SNP loci using the MassARRAY markers showed that all of the nR radishes had C, A, and A, whereas R radishes had A, T, and G for alleles 1, 2, and 3, respectively (**Figure 2B**; **Table 1**). Protein structure prediction with AlphaFold DB showed that amino acid changes due to nonsynonymous SNPs may affect C-terminal structure of the RsMYB1.1 proteins (**Supplementary Figure S1**).

**Table 1:** Genotypes of SV and SNP loci in the promoter and coding sequences of *RsMYB1*.*1* genes of core collection accessions and commercial cultivars.

Type	Name	Source	Root skin color	SV^a^ in	SNP^b^ in exon 3		
				promoter	allele 1	allele 2	allele 3
Core collection	WK10039	NIHHS^c^, Korea	white	Insertion	C	A	A
	CUR003	CUK^d^, Korea	white	Insertion	C	A	A
	CUR008	CUK, Korea	white	Insertion	C	A	A
	CUR010	CUK, Korea	white	Insertion	C	A	A
	CUR025	CUK, Korea	white	Insertion	C	A	A
	CUR037	CUK, Korea	white	Insertion	C	A	A
	CUR046	CUK, Korea	white	Insertion	C	A	A
	CUR048	CUK, Korea	white	Insertion	C	A	A
	CUR058	CUK, Korea	white	Insertion	C	A	A
	WK10024	NIHHS, Korea	red	Deletion	A	T	G
	CUR009	CUK, Korea	red	Deletion	A	T	G
	CUR022	CUK, Korea	red	Deletion	A	T	G
	CUR034	CUK, Korea	red	Deletion	A	T	G
	CUR076	CUK, Korea	red	Deletion	A	T	G
	CUR080	CUK, Korea	red	Deletion	A	T	G
	CUR082	CUK, Korea	red	Deletion	A	T	G
	CUR084	CUK, Korea	red	Deletion	A	T	G
	CUR085	CUK, Korea	red	Deletion	A	T	G
Commercial cultivars	Baeg-un mu	Nongwoobio, Korea	white	Insertion	C	A	A
	Chammat	Syngenta, Korea	white	Insertion	C	A	A
	Cheongdam mu	Kyoungshin Seed Co., Korea	white	Insertion	C	A	A
	Cheongsu mu	Dongwon Nongsan Seed, Korea	white	Insertion	C	A	A
	Chunchujangbaeg mu	Danong, Korea	white	Insertion	C	A	A
	Danhong mu	Asia Seed, Korea	white (red flesh)	Insertion	C	A	A
	Eomji mu	Green Heart Bio, Korea	white	Insertion	C	A	A
	Gegeol mu	Hyundae Seed Co., Korea	white	Insertion	C	A	A
	Geumjeong Spring mu	Kyoungshin Seed Co., Korea	white	Insertion	C	A	A
	Gwail mu	Jinhung Seed, Korea	white (red flesh)	Insertion	C	A	A
	Huindung-i mu	Asia Seed, Korea	white	Insertion	C	A	A
	Jinju Daepyeong mu	Asia Seed, Korea	white	Insertion	C	A	A
	Minong mu	Asia Seed, Korea	white	Insertion	C	A	A
	Saessag red mu	CNSEED, Korea	white	Insertion	C	A	A
	Sunbaeg-og mu	Danong, Korea	white	Insertion	C	A	A
	Taecheong mu	FarmHannong, Korea	white	Insertion	C	A	A
	Asiateuk mu^e^	Asia Seed, Korea	red	Deletion	A	T	G
	Comet^e^	Asia Seed, Korea	red	Deletion	A	T	G
	Plum purple^e^	Lake Valley Seed, USA	red	Deletion	A	T	G
	Champion^e^	Lake Valley Seed, USA	red	Deletion	A	T	G
	Sparker^e^	Lake Valley Seed, USA	red	Deletion	A	T	G
	Jeoghwan 20-day radish^e^	Kyoungshin Seed Co., Korea	red	Deletion	A	T	G

a SV, Structural Variation.

b SNP, Single Nucleotide Polymorphism.

c NIHHS, National Institute of Horticultural and Herbal Science.

d CUK, The Catholic University of Korea.

e 20-day radish cultivars.

**Figure 3:**
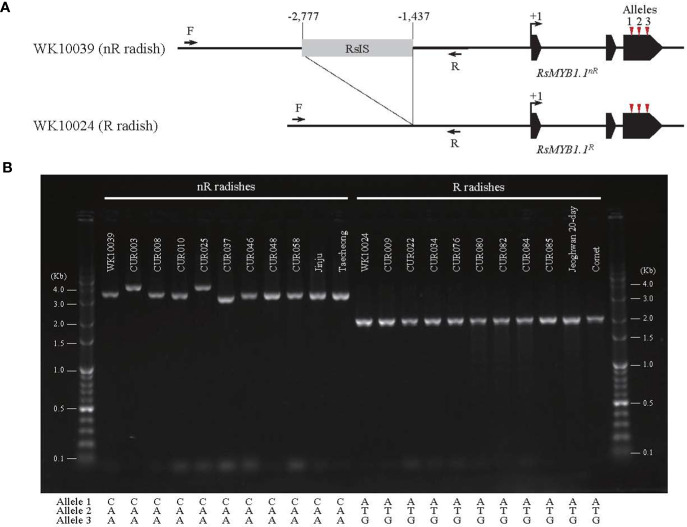
The PAV in the promoter region of *RsMYB1*.*1*. **(A)** The 1.3 kb RsIS is inserted 1.4 kb upstream from the start codon of *RsMYB1*.*1* of WK10039; the corresponding region of WK10024 has no RsIS. Arrows indicate primers for PCR amplification. Numbers are upstream positions from the start codon (+1). **(B)** Agarose gel electrophoresis of PCR amplicons from the promoter region of *RsMYB1*.*1* of radish accessions. Only the non-red-skinned (nR) radishes have RsIS insertions with length variation. The positions and sequences of nonsynonymous SNP alleles for each accession are also shown.

The promoter region of *RsMYB1*.*1* showed group-specific SVs. We extracted the promoter region (approximately 4 kb in length from the start codon) of each *RsMYB1*.*1* gene. Multiple alignments of the sequences showed that the nR radishes had an insertion sequence of average size 1.3 kb in the upstream 1.4 kb region from the start codon of *RsMYB1*.*1* (–2777 to –1437 bp of *RsMYB1*.*1* in WK10039). By contrast, the R radishes had no insertion sequences in the corresponding promoter regions (**Supplementary Figure S2**). This InDel variation was also detected in genome assemblies (Zhang et al., 2021), in which seven genotypes with white- or black-skinned taproots had insertions but only Rs06 (red-skinned taproot) had a deletion in the promoter region (**Supplementary Figure S3**). Agarose gel electrophoresis of PCR amplicons from the promoter region of *RsMYB1*.*1* demonstrated that the nR radishes had 1.3 to 2 kb insertions in the target region, which was absent in all of the R radishes, indicating a PAV of SV (**Figure 3**). We named it the *R*. *sativus* insertion sequence (RsIS).

### 3.3 RsIS is an AT-rich sequence distributed throughout the radish genome

The RsIS of WK10039 was highly rich in AT (78.7% AT content). The RsIS is a low-complexity DNA sequence of a biased composition containing many simple sequences repeat units. It contained 167 repeat units of 11 to 21 nucleotides and a number of predicted secondary hairpin structures (**Supplementary Figure S4**). We analyzed the sequence using the Plant Repeat Database, however, neither specific matches to the known repeat elements including DNA transposons, retroelements, and long terminal repeats, nor repeated patterns were identified. A BLASTN search (cutoff of 1E^–10^ and query and subject coverages of 50%) of the RsIS of WK10039 against radish, diploid *Brassica*, and *Arabidopsis* genomes revealed that this sequence was specifically amplified in both the cultivar and wild radish genomes and had CNVs (132 to 183 copies) (**Supplementary Table S5**). RsIS had 144 BLAST matches distributed on nine chromosomes of WK10039. Sequence analysis revealed that RsIS consists of two regions. The front 700 bp is a conserved region between the BLAST matched sequences, showing 74.4–85.3% nucleotide identity, and the rear 600 bp is a variable region with <50% nucleotide identity. Most of the RsIS-like sequences (60%) were in intergenic regions, 43 copies were in the promoter regions (up to 3 kb upstream of genes) of 43 genes, and 15 copies were in the introns of 14 genes (**Supplementary Table S6**). Interestingly, mRNA-seq analysis showed that the expression levels of the genes harboring RsIS-like sequences in their promoter regions were low in a variety of tissues (**Supplementary Table S7**). The average TMM values of 41 of 43 genes were <50. The transcription of *RsMYB1*.*1* was markedly downregulated in all the investigated tissues of WK10039 with an average TMM value of 1.2.

### 3.4 *RsMYB1*.*1* and genes encoding components of the anthocyanin biosynthesis pathway are downregulated in the root of non-red-skinned radishes

**Figure 4:**
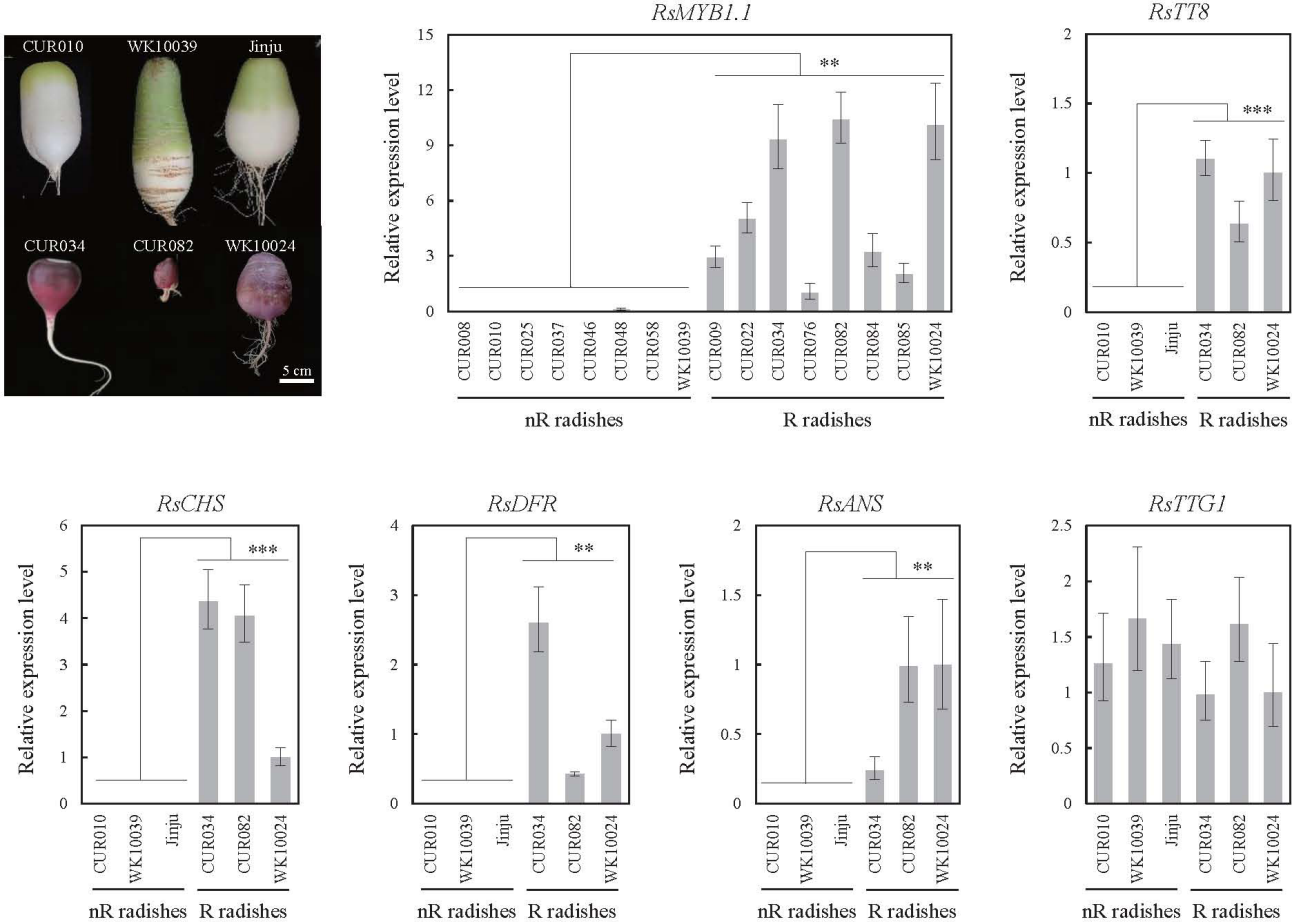
Expression levels of the MBW complex (*RsMYB1*.*1*, *RsTT8*, and *RsTTG1*) and selected structural genes (*RsCHS*, *RsDFR*, and *RsANS*) of the anthocyanin biosynthesis pathway in the radish accessions as determined by qPCR. Root tissues were harvested 42 days after germination. Comparative cycle threshold (2^–ΔΔCt^) values represent relative expression calculated using the CUR076 (*RsMYB1*.*1*) and WK10024 (other genes) samples as a reference. Error bars depict the standard deviations of three independent biological replicates. Asterisks represent statistical significance (^**^p < 0.01, ^***^p < 0.001) between nR and R radishes by t-test.

We examined the expression profiles of three key transcription factors (RsMYB1.1, R9.018870 encoding RsTT8, and R4.018220 encoding RsTTG1) of the MBW complex and three anthocyanin biosynthesis genes (R2.030580 encoding RsCHS, R9.040290 encoding RsDFR, and R2.039640 encoding RsANS) in the roots of nR and R radishes by qPCR (**Figure 4**). The expression levels of *RsMYB1*.*1* were significantly lower in the eight nR radishes than in the eight R radishes (p < 0.01). Insertion of the RsIS in the promoter region of *RsMYB1*.*1* likely suppresses the transcription of *RsMYB1*.*1* in nR radishes. Considering the stability of root character, the availability of sufficient seeds for experiments, and available genome sequences, we selected three nR radishes (CUR010, WK10039, and Jinju) and three R radishes (CUR034, CUR082, and WK10024) for further analysis. Similar to *RsMYB1*.*1*, *RsTT8*, *RsCHS*, *RsDFR*, and *RsANS* were expressed only in the R radishes. By contrast, *RsTTG1* was expressed at similar levels in the nR and R radishes. These results are consistent with the anthocyanin levels of the nR and R radishes in the core collection accessions (Huh et al., 2023); therefore, downregulation of the anthocyanin biosynthesis pathway genes in the nR radishes reduces the anthocyanin accumulation in the root, resulting in non-red-colored skin. The downregulation of not only *RsMYB1*.*1* but also *RsTT8* may be involved in the suppression of anthocyanin biosynthesis in the root of nR radishes.

**Figure 5:**
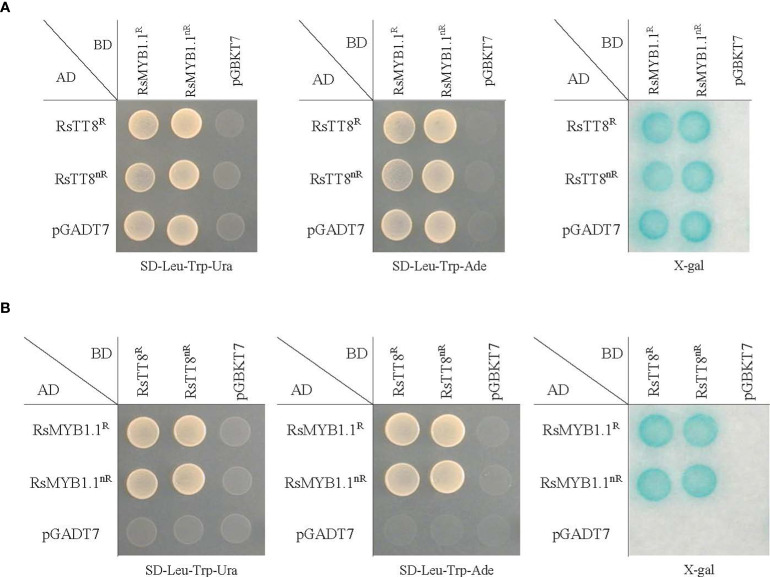
Pairwise interaction between RsMYB1.1 and RsTT8 in the yeast two-hybrid (Y2H) assay. The prey vector pGADT7 (AD) and the bait vector pGBKT7 (BD) are Y2H vectors without an insert. *RsMYB1*.*1* and *RsTT8* of WK10039 (nR) and CUR034 (R) were cloned into pGADT7 and pGBKT7. PBN204 yeast was cotransformed with the bait (BD) and prey (AD) vectors. Interactions between the two proteins were identified in both cases using RsMYB1.1 as the bait and RsTT8 as the prey **(A)** and RsMYB1.1 as the prey and RsTT8 as the bait **(B)**.

To examine the physical interaction between RsMYB1.1 and RsTT8, we performed a pairwise yeast two-hybrid (Y2H) assay (**Figure 5**). The synthesized full-length CDSs of *RsMYB1*.*1* and *RsTT8* of WK10039 (nR radish; *RsMYB1*.*1^nR^* and *RsTT8^nR^*) and CUR034 (R radish; *RsMYB1*.*1^R^* and *RsTT8^R^*) were cloned into bait pGBKT7 and prey pGADT7 vectors. The identities of the two proteins between WK10039 and CUR034 were 98.0% (RsMYB1.1) and 98.5% (RsTT8). The bait and prey vectors were cotransformed into PBN204 yeast, which was cultured on synthetic dropout medium lacking leucine, tryptophan, and uracil or adenine. Pairwise coexpression of RsMYB1.1 and RsTT8 of WK10039 and CUR034 led to the growth of yeast colonies, indicating that RsMYB1.1 of WK10039 and CUR034 interact with RsTT8 of WK10039 and CUR034 and vice versa. A filter assay confirmed the interactions among these proteins. Both RsMYB1.1 of WK10039 and CUR034 showed self-transcriptional activation but RsTT8 did not. RsMYB1.1 and RsTT8 showed a similar interaction in AH109 yeast (data not shown). Therefore, RsMYB1.1 of nR and R radishes may contribute similarly to the formation of the MBW complex. Nevertheless, the downregulation of *RsMYB1*.*1* likely suppresses anthocyanin biosynthesis in nR radishes.

### 3.5 RsMYB1.1 proteins of non-red- and red-skinned radishes function similarly in anthocyanin biosynthesis

**Figure 6:**
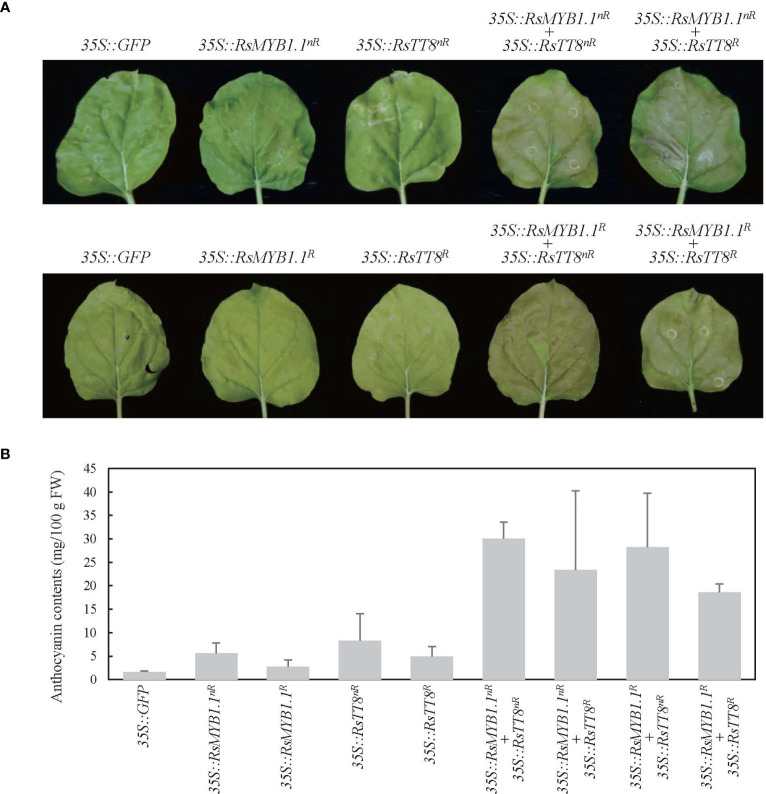
Transient expression of *RsMYB1*.*1* and *RsTT8* in tobacco leaves. **(A)** *RsMYB1*.*1* and *RsTT8* of WK10039 (nR) and CUR034 (R) under the control of the 35S promoter were expressed individually or coexpressed in *N*. *benthamiana* leaves. Photographs were obtained 5 days after *Agrobacterium* infiltration. **(B)** Total anthocyanin levels in *Agrobacterium-*infiltrated tobacco leaves. Error bars depict the standard deviations of three independent biological replicates.

To determine whether the RsMYB1.1 proteins of nR and R radishes are functionally active in anthocyanin biosynthesis, we expressed CDSs of *RsMYB1*.*1^nR^* and *RsMYB1*.*1^R^* individually or together with *RsTT8^nR^* or *RsTT8^R^* in *N*. *benthamiana* leaves under the control of the 35S promoter and measured the anthocyanin contents 5 days after *Agrobacterium* infiltration (**Figure 6**). Transient overexpression of individual RsMYB1.1 or RsTT8 hardly changed leaf color and led to an accumulation of a small amount of anthocyanin (2.7–8.3 mg/100 g FW) in tobacco leaves, whereas expression of the nR radish genes resulted in slightly greater accumulation of anthocyanins than the R radish genes. Coexpression of RsMYB1.1^nR^ with RsTT8^nR^ resulted in a pale-red leaf and the highest accumulation of anthocyanins (30.1 mg/100 g FW), which was approximately 5.3- and 1.6-fold higher than caused by the expression of RsMYB1.1^nR^ and coexpression of RsMYB1.1^R^ with RsTT8^R^, respectively. Interestingly, the combinations of RsMYB1.1^nR^ with RsTT8^R^ and RsMYB1.1^R^ with RsTT8^nR^ also increased the accumulation of anthocyanins (23.4–28.3 mg/100 g FW) compared to the expression of RsMYB1.1 or RsTT8 individually. Coexpression of RsMYB1.1^nR^ with RsTT8^nR^ also increased anthocyanin accumulation in WK10039 and *N*. *tabacum* leaves (**Supplementary Figure S5**). Therefore, RsMYB1.1^nR^ has no functional defect compared to RsMYB1.1^R^ despite three different amino acid residues as a result of SNPs in its CDS, and coexpression of RsMYB1.1 with RsTT8 increased anthocyanin biosynthesis. The proposed role of RsIS in the promoter region of *RsMYB1*.*1^nR^* needs to be confirmed by expressing genes under the control of the native promoter.

### 3.6 The RsIS negatively regulates the *RsMYB1.1* promoter in non-red-skinned radish

**Figure 7:**
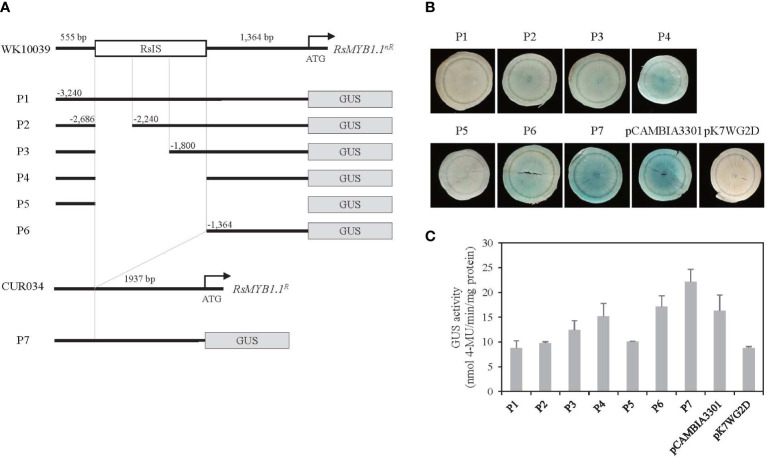
Histochemical analysis of transient GUS expression in radish taproot section. **(A)** Schematics of *RsMYB1*.*1* promoter::GUS fusion constructs. P1 to P6 are successive deletion constructs for *pRsMYB1*.*1^nR^* of WK10039. P7 is a construct for *pRsMYB1*.*1^R^* of CUR034. The white box is the RsIS in *pRsMYB1*.*1* of WK10039 and the gray boxes are the GUS coding regions. ATG is the start codon. Numbers are upstream positions from the start codon. **(B)** Photographs of GUS-stained WK10039 taproot sections obtained 3 days after *Agrobacterium* infiltration. GUS staining was performed for 24 h. **(C)** GUS activity expressed as nmol 4-methyl-umbelliferone min^–1^ mg^–1^ soluble protein. Error bars depict the standard deviations of three independent biological replicates.

The function of the RsIS in *RsMYB1*.*1^nR^* expression was investigated following the transient transformation of radish root sections by *Agrobacterium* infiltration. To determine whether the RsIS negatively regulates the activity of the *RsMYB1*.*1^nR^* promoter, we cloned the promoters of *RsMYB1*.*1^nR^* and *RsMYB1*.*1^R^* and generated seven promoter::GUS fusion constructs (**Figure 7A**). The P1 to P4 constructs included the 3.2 kb native promoter of *RsMYB1*.*1^nR^* and successive deletions of the RsIS region. The P5 and P6 constructs contained 5’-upstream 0.6 kb and 3’-downstream 1.4 kb fragments of the *RsMYB1*.*1^nR^* promoter, respectively. The P7 construct has the 1.9 kb native promoter of *RsMYB1*.*1^R^*, which corresponds to P1 except for the RsIS. Three independently transformed root sections of WK10039 were examined to analyze the effect of the RsIS on gene expression.

No GUS expression was detected in the P1 and P5 constructs whereas strong GUS staining was observed in the P7 construct, indicating that the promoter of *RsMYB1*.*1^nR^* is inactive (**Figure 7B**). However, the expression of *RsMYB1*.*1^nR^* promoter::GUS was altered by deletion of the RsIS. Very faint GUS staining was detected in the P2 construct, in which one third of the RsIS was deleted. The P3 construct, in which two thirds of the RsIS was deleted, showed slightly increased GUS staining compared to the P2 construct. Moreover, distinct staining was observed in the P4 construct, in which the whole RsIS was deleted. Similar GUS staining was detected in the P6 construct, which consisted of 1.4 kb of the core promoter of *RsMYB1*.*1^nR^* lacking the RsIS. A fluorometric GUS assay indicated that deletion of RsIS from the promoter of *RsMYB1*.*1^nR^* (P4 and P6) enhanced GUS expression approximately 1.7- to 2.0-fold relative to P1, which reached 69–77% of the expression level of *RsMYB1*.*1^R^* (P7) (**Figure 7C**). Therefore, deletion of the RsIS was correlated with increased expression of the GUS reporter gene, indicating that the RsIS negatively regulates the promoter activity of *RsMYB1*.*1^nR^*.

## 4 Discussion

Unraveling the genetic basis of agronomic traits is a challenge in agriculture and crop science. Advances in genome sequencing techniques and the availability of an increasing number of quality reference genome assemblies have enabled GWASs with the aim of discovering novel genes in diverse crop species. We identified a genetic element that negatively regulates the skin color of radish taproot by performing an InDel GWAS using the Rs2.0 genome assembly, high-depth resequencing data, and phenotype data of 100 radish accessions. We identified a highly variable region in the promoter of *RsMYB1*.*1* on the R2 chromosome. Although we detected three nonsynonymous SNPs in the third exon of *RsMYB1*.*1* between nR and R radishes, the RsMYB1.1 proteins of nR and R radishes had no functional differences. This suggests that nonsynonymous SNPs in the coding sequence of *RsMYB1*.*1* are likely to have arisen as a result of selective sweep during breeding of cultivars. By contrast, insertion of 1.3 kb of the AT-rich RsIS in the promoter region of *RsMYB1*.*1^nR^* significantly downregulated its expression, suppressing anthocyanin biosynthesis and the growth of nR radishes. Successive deletions of RsIS from promoter sequences indicated that its removal restores the activity of the *RsMYB1*.*1^nR^* promoter. Therefore, this type of PAV could be the genetic basis of the phenotypic difference between nR and R radishes.

The sizes of plant genomes tended to increase during evolution as a result of, for example, occasional polyploidy and the accumulation of repetitive sequences. In the tribe Brassiceae of the family Brassicaceae, radish and its closely related diploid *Brassica* species (*B*. *rapa*, *B*. *nigra*, and *B*. *oleracea*) underwent triplication of their entire genomes, resulting in genomes approximately 4.2- to 4.6-fold larger than *A*. *thaliana*. Differential lineage-specific amplification of repetitive sequence elements is likely to be responsible for the variation in size and function in the radish and *Brassica* genomes. Radish arose from the *Brassica* B genome lineage and is a sibling species of *B*. *nigra* rather than *B*. *rapa* and *B*. *oleracea* (Cho et al., 2022). The most important differences between the radish and *B*. *nigra* genomes are the proportions of repetitive sequences and uniquely amplified elements. The repetitive sequence fraction of the radish genome is 38%, which is smaller than that of *B*. *nigra* (49–54%) but similar to those of *B*. *rapa* (38%) and *B*. *oleracea* (39%). Although the predominant repetitive sequences in the radish and *B*. *nigra* genomes are Ty3/Gypsy retrotransposons, Ale family Ty1/Copia retrotransposons have been amplified in the chromosomes and centromeres of *B*. *nigra* as a result of recent nested transposition events (Perumal et al., 2020). In this study, a low-complexity sequence, RsIS, was amplified only from the radish genome. Based on the number of short repeat units in the RsIS, we hypothesize that replication slippage or slipped-strand mispairing may have resulted in misalignment of DNA strands during the replication of short, repeated DNA sequences, leading to the emergence of the RsIS. Chromosome rearrangement, homologous exchange, and unequal crossing-over in the radish lineage after splitting of the radish and *B*. *nigra* genomes 11.1 Mya may have contributed to the distribution of the RsIS throughout the chromosomes of radish.

Lineage-specific amplification of repetitive sequences can be a major source of insertions, resulting in PAVs with profound effects on phenotypic and genomic variation. In the sorghum genome, the major components of PAVs are mobile elements and repeat sequences (Zhang et al., 2014). Insertion of TEs can affect the expression of nearby genes, and regulate gene function in diverse ways such as *cis* up- or downregulation of transcription by insertion at the promoter, exon, intron, and downstream regions, and *trans* production of short interfering RNAs (siRNAs) via two RNA-directed DNA methylation pathways (Gill et al., 2021). Similarly, the insertion of low-complexity AT-rich repetitive sequences may modify the expression of nearby genes by altering the promoter, coding, and downstream regions or the topology of the chromatin strand. We detected CNVs (132 to 183 copies) of RsIS among the radish genomes sequenced to date. Identification of RsIS as a PAV indicates that several RsIS diverged as low-frequency PAVs, leading to novel variants and accelerating the generation of new genetic elements that negatively regulate phenotypic traits. These results are consistent with the findings of a radish pan-genome study, suggesting that insertion is more important than deletion in the SVs in *Raphanus* genomes (Zhang et al., 2021). Because AT-rich DNA is concentrated in the nucleosome-free regions (NFRs) associated with the transcription start sites of most genes (Lorch et al., 2014), RsIS may disturb the transcription of *RsMYB1*.*1^nR^* by acting as a redundant NFR at the wrong position or changing the topology of the chromatin strand. Additionally, AT-rich sequences are known to interact with regulatory proteins and especially non-coding RNAs involved in transcriptional regulation (Kato et al., 2023). These interactions can in turn fine-tune gene expression levels in response to cellular signals. Further study is needed to determine whether RsIS influences the transcription of genes or the conformation of chromatin.

*RsMYB1* is a radish ortholog of *AtPAP1* (AT1G56650) which encodes a key transcription factor involved in anthocyanin accumulation in *A*. *thaliana*. Three or four *RsMYB1* paralogs have been identified on the R2, R5, and R7 chromosomes of radish. *RsMYB1*.*1* (R2.009390) is one of three *RsMYB1* paralogs in the genome of WK10039; the others are *RsMYB1*.*2* (R5.050800) and *RsMYB1*.*4* (R7.017240); *RsMYB1*.*3*, which was identified as Rsa10033919 on the R7 chromosome of cv. XYB36–2 (Liu et al., 2019a), was absent in the WK10039 genome. In the cv. XYB36–2 genome, *RsMYB1*.*3* and *RsMYB1*.*4* are separated by approximately 1.97 Mb on the R7 chromosome and are more closely related than the other two paralogs (**Supplementary Figure S6**). To date, three *RsMYB1* paralogs (*RsMYB1*.*1*, *RsMYB1*.*3*, and *RsMYB1*.*4*) have been suggested as candidate genes related to anthocyanin biosynthesis in radish, however, which is the key determinant of red taproot depends on the accessions analyzed. QTL-Seq combined with linkage analysis suggested *RsMYB1*.*1* as a candidate gene for the purple skin of the taproot of cv. CX16Q-25–2, however, the function of this gene and its transcriptional regulation are unclear (Liu et al., 2019a). *RsMYB1*.*3* reportedly regulates anthocyanin biosynthesis in cv. Bordeaux (Lim et al., 2016) and cv. Xinlimei (Liu et al., 2019b), both of which have taproots with red flesh. The involvement of *RsMYB1*.*4* in anthocyanin synthesis has been reported in the red-skinned radish cv. Lian Yan No. 1 (Yi et al., 2018). In addition, QTL-seq and transcriptome sequencing of the red-fleshed radish cv. MTH01 (Wang et al., 2020) and a molecular genetic study of the red-skinned radish cv. HongPi (Kim et al., 2021) have identified *RsMYB1*.*4*. Interestingly, a short insertion in the exon of *RsMYB1*.*3* and epigenetic regulation of the promoter region of *RsMYB1*.*4* disrupts anthocyanin accumulation. Because *RsMYB1*.*1* and *RsMYB1*.*3/RsMYB1*.*4* are located on different chromosomes and are separated into distinct phylogenetic clades, we hypothesize that *RsMYB1*.*1* and *RsMYB1*.*3/RsMYB1*.*4* have different functions in the tissues of the radish taproot. We propose that the function of *RsMYB1*.*1* is restricted to the skin, compared to the flesh and skin for *RsMYB1*.*3/RsMYB1*.*4*. This suggests that the *RsMYB1* paralog that regulates anthocyanin synthesis in radish taproot is genotype dependent. It is important to categorize radish genetic resources according to the expression of *RsMYB1* paralogs to enable targeted breeding of radish.

Anthocyanin biosynthesis is a multistep process transcriptionally regulated by the MBW complex, which interacts with MYB- and bHLH-recognition elements in the promoter regions of anthocyanin biosynthesis genes (Zhu et al., 2015). Our findings show that the expression of *RsMYB1*.*1* must be coordinated with that of *RsTT8* for the accumulation of anthocyanins in radish and tobacco tissues. Irrespective of what nR and R radish lines the two proteins originated from, coexpression of *RsMYB1*.*1* and *RsTT8* is necessary for anthocyanin biosynthesis; overexpression of each gene individually did not result in anthocyanin biosynthesis. Given the lack of *RsTT8^nR^* expression in the taproots of nR radishes and the absence of any structural variation in the promoter, CDS, and downstream region of *RsTT8* between nR and R radishes, it is probable that the transcriptional regulation of *RsTT8^nR^* is different from that of *RsMYB1*.*1^nR^*. According to previous studies of transcription factor genes in anthocyanin pathways (Oikawa et al., 2015; Zhang et al., 2019; Wang et al., 2020; Yang et al., 2021), remote transcriptional regulators, post-transcriptional regulation, or epigenetic mechanisms may influence the expression of *RsTT8^nR^*. Genome-wide miRNA sequencing or estimation of the degree of methylation of *RsTT8^nR^* and its flanking sequences by McrBC-PCR and bisulfite sequencing of nR radish will provide insight into the regulatory mechanism of *RsTT8^nR^* expression. Our hybridization experiment of radish accessions with red-skin/white-flesh taproots and white-skin/red-flesh taproots demonstrated that the red-skin color is inherited as a single dominant trait whereas red flesh is unstable and maternally influenced (unpublished data). Therefore, RsTT8 may act as a maternal regulator of flesh color. In *A*. *thaliana*, fatty acid accumulation in seeds and triploid seed development are maternally controlled by *TT8* (Chen et al., 2014; Zumajo-Cardona et al., 2023). Characterization of the functions of *RsTT8* and *RsMYB1*.*1* may enable the development of a method to control taproot pigmentation for use in radish breeding programs.

## Data availability statement

The original contributions presented in the study are included in the article/**Supplementary Material**, further inquiries can be directed to the corresponding authors.

## Author contributions

JK: Formal Analysis, Resources, Writing – original draft. HJ: Data curation, Formal Analysis, Methodology, Software, Writing – review & editing. SH: Data curation, Resources, Writing – review & editing. AC: Data curation, Resources, Software, Validation, Visualization, Writing – review & editing. BY: Methodology, Resources, Writing – review & editing. S-HJ: Data curation, Resources, Writing – review & editing. HK: Data curation, Resources, Writing – review & editing. H-JY: Conceptualization, Data curation, Funding acquisition, Project administration, Supervision, Validation, Writing – original draft, Writing – review & editing. J-HM: Conceptualization, Data curation, Funding acquisition, Project administration, Supervision, Validation, Writing – original draft, Writing – review & editing.
