# Supplementary material for: Effect of structural variation in the promoter region of RsMYB1.1 on the skin color of radish taproot
Source: Front Plant Sci. 2024 Jan 8;14:1327009. doi: 10.3389/fpls.2023.1327009 (PMC10804855; doi:10.3389/fpls.2023.1327009)
Supplement: Supplementary file 1 [file Presentation_1.pptx]

## Slide 1
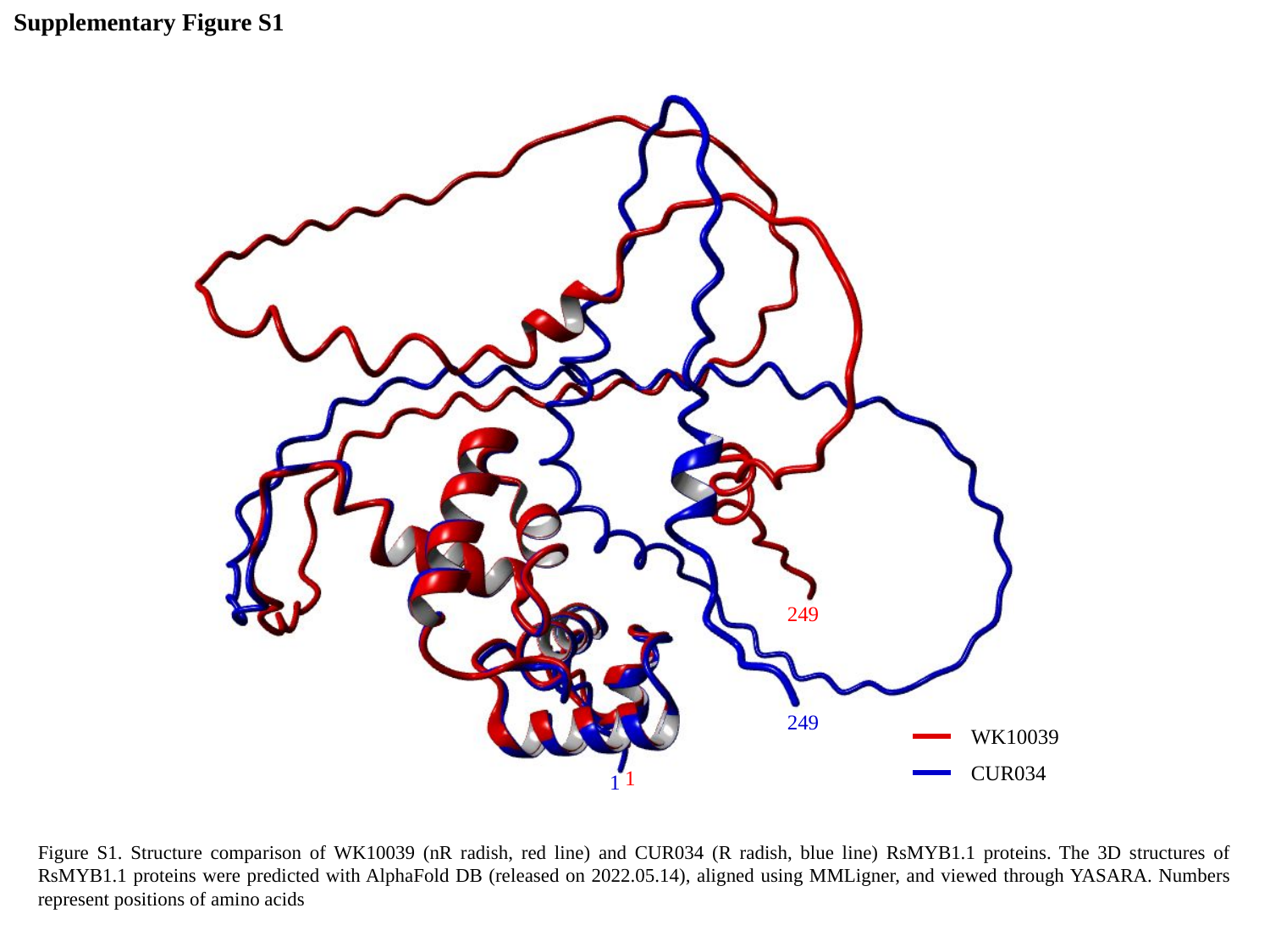

Supplementary Figure S1
249
249
1
1
WK10039
CUR034
Figure S1. Structure comparison of WK10039 (nR radish, red line) and CUR034 (R radish, blue line) RsMYB1.1 proteins. The 3D structures of RsMYB1.1 proteins were predicted with AlphaFold DB (released on 2022.05.14), aligned using MMLigner, and viewed through YASARA. Numbers represent positions of amino acids

## Slide 2
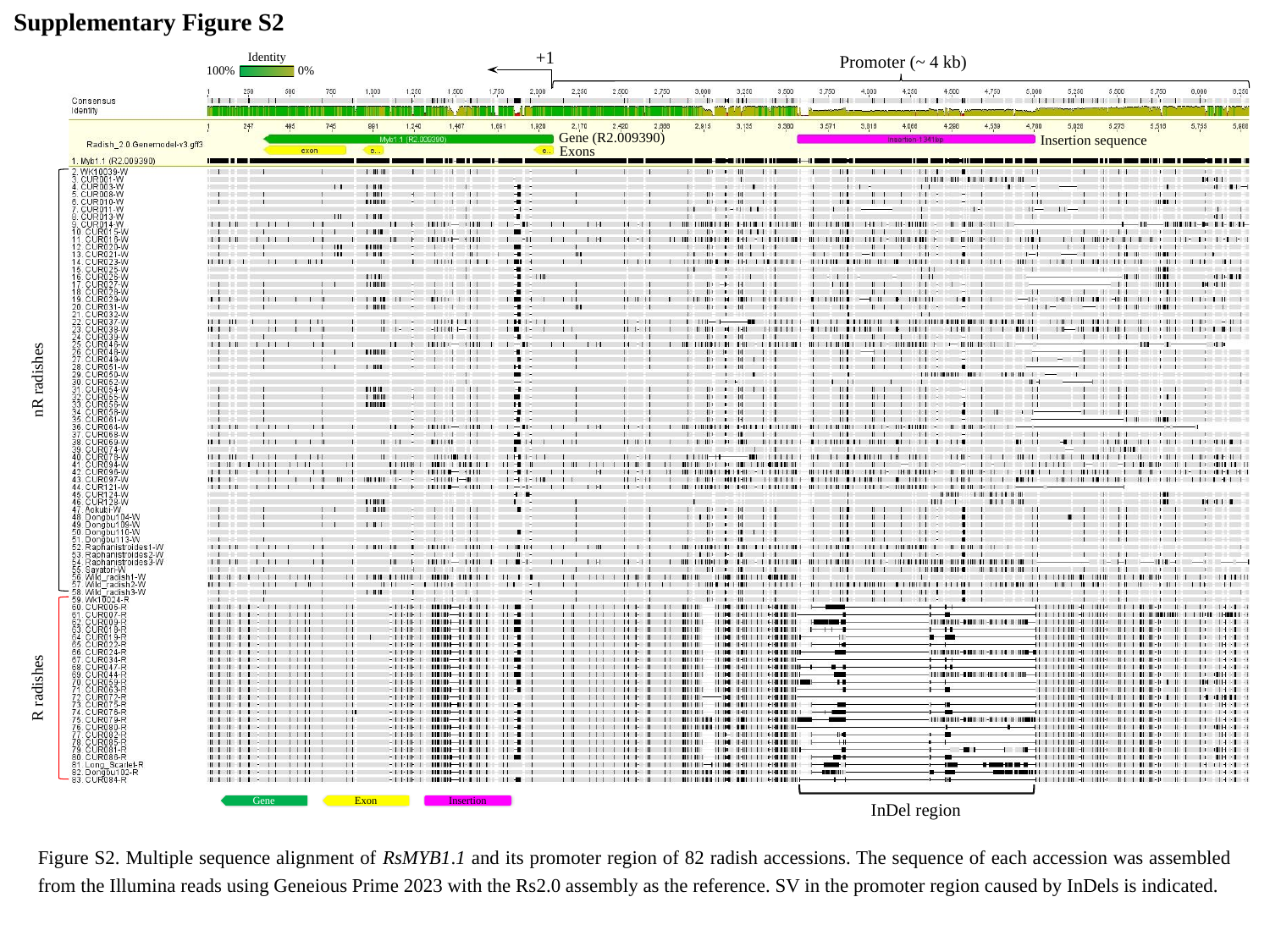

Supplementary Figure S2
+1
Promoter (~ 4 kb)
nR radishes
R radishes
InDel region
Identity
100%
0%
Gene (R2.009390)
Insertion sequence
Exons
Gene
Exon
Insertion
Figure S2. Multiple sequence alignment of RsMYB1.1 and its promoter region of 82 radish accessions. The sequence of each accession was assembled from the Illumina reads using Geneious Prime 2023 with the Rs2.0 assembly as the reference. SV in the promoter region caused by InDels is indicated.

## Slide 3
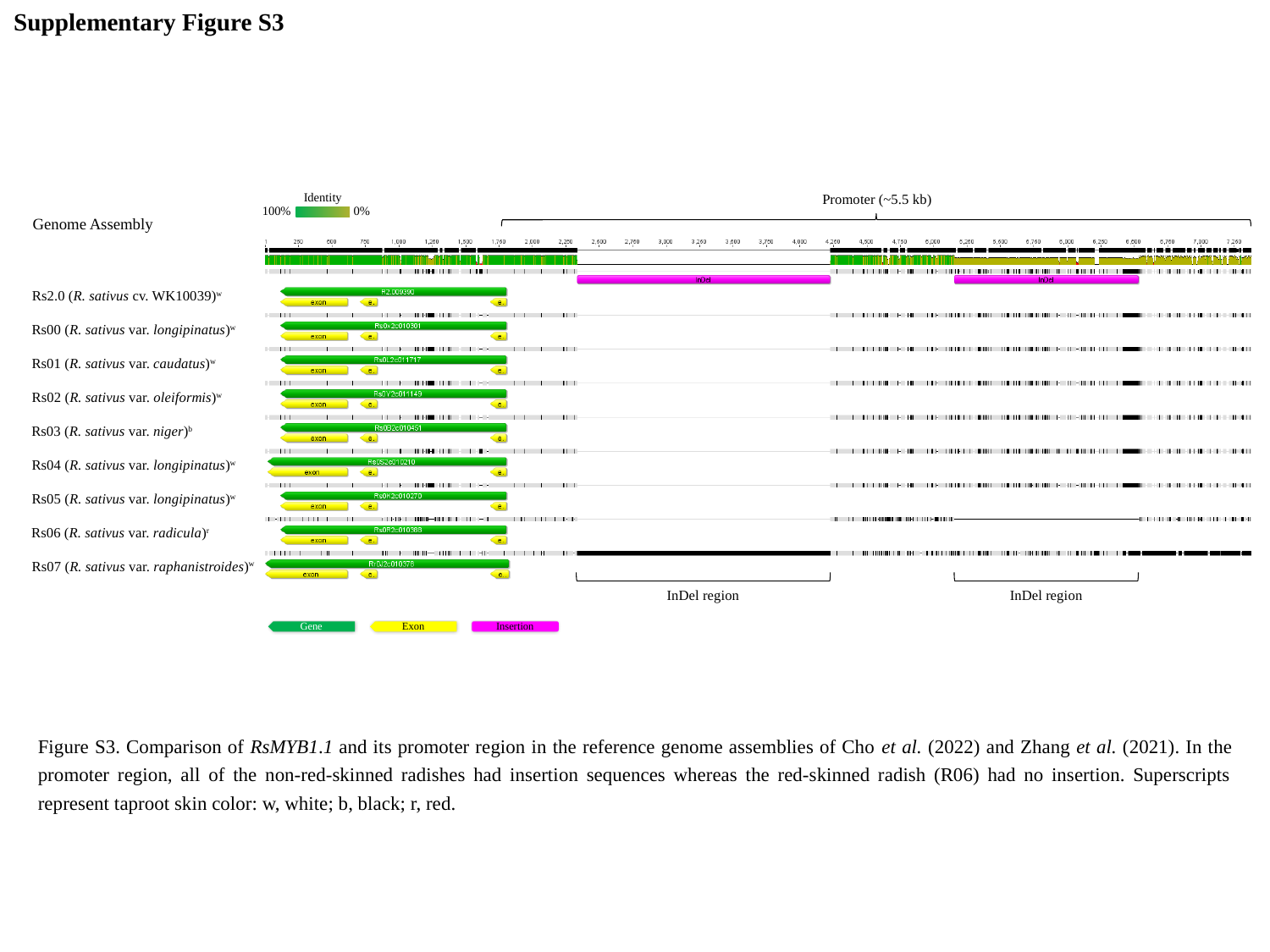

Supplementary Figure S3
Identity
100%
0%
Promoter (~5.5 kb)
InDel region
InDel region
Genome Assembly
Rs2.0 (R. sativus cv. WK10039)w
Rs00 (R. sativus var. longipinatus)w
Rs01 (R. sativus var. caudatus)w
Rs02 (R. sativus var. oleiformis)w
Rs03 (R. sativus var. niger)b
Rs04 (R. sativus var. longipinatus)w
Rs05 (R. sativus var. longipinatus)w
Rs06 (R. sativus var. radicula)r
Rs07 (R. sativus var. raphanistroides)w
Gene
Exon
Insertion
Figure S3. Comparison of RsMYB1.1 and its promoter region in the reference genome assemblies of Cho et al. (2022) and Zhang et al. (2021). In the promoter region, all of the non-red-skinned radishes had insertion sequences whereas the red-skinned radish (R06) had no insertion. Superscripts represent taproot skin color: w, white; b, black; r, red.

## Slide 4
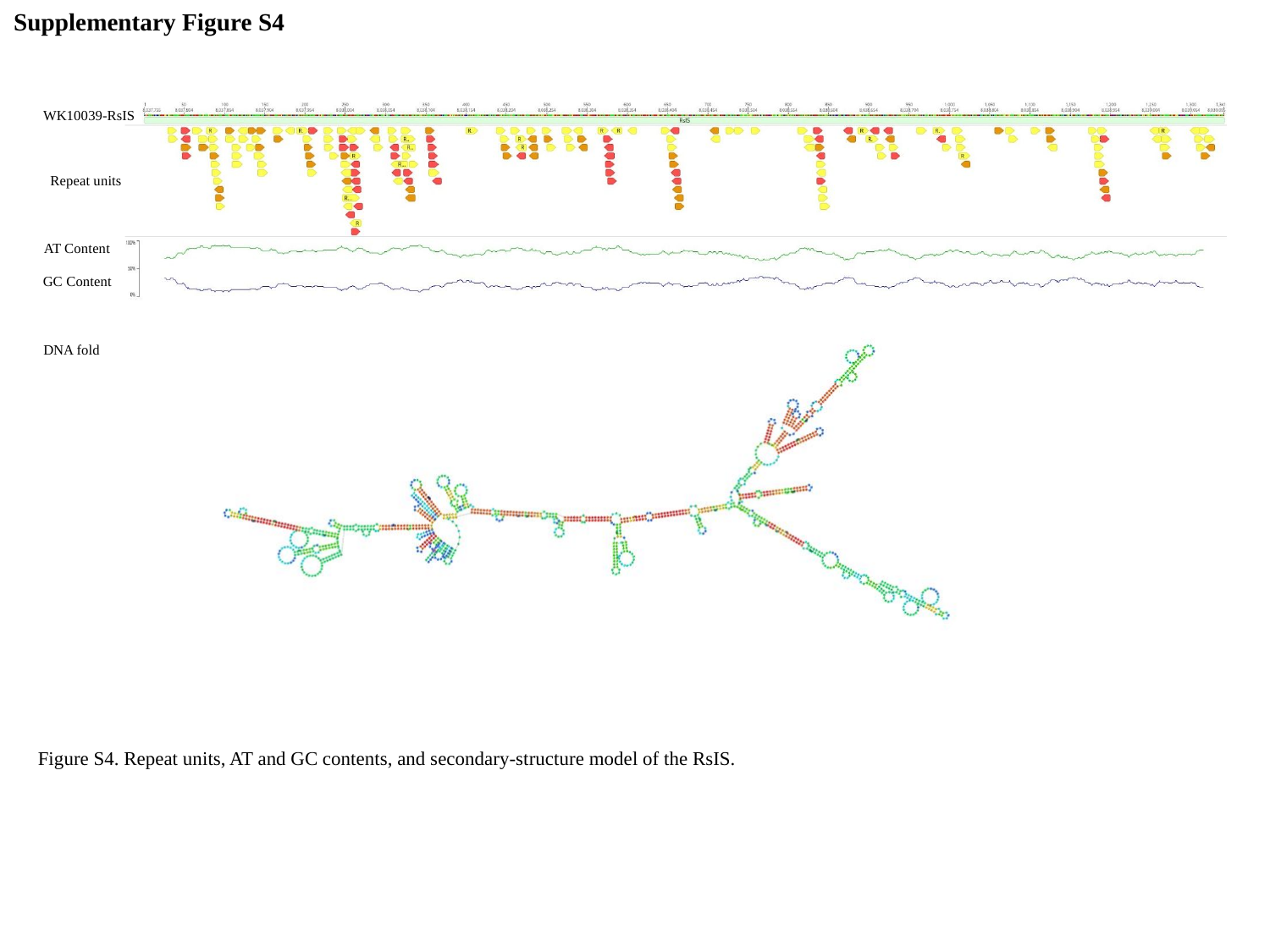

Supplementary Figure S4
WK10039-RsIS
Repeat units
AT Content
GC Content
DNA fold
Figure S4. Repeat units, AT and GC contents, and secondary-structure model of the RsIS.

## Slide 5
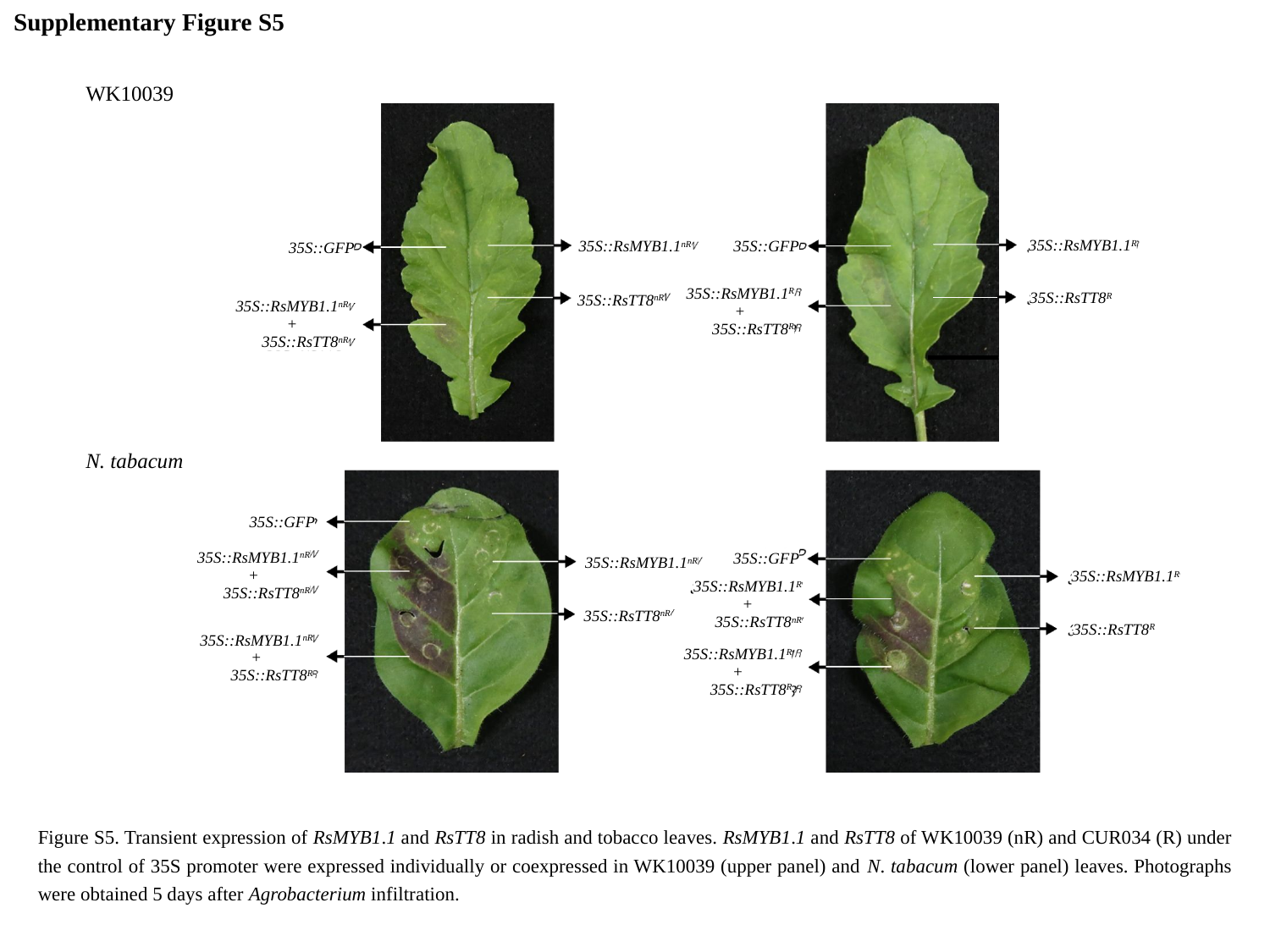

Supplementary Figure S5
WK10039
35S::RsMYB1.1R
35S::RsMYB1.1nR
35S::GFP
35S::GFP
35S::RsMYB1.1R
+
35S::RsTT8R
35S::RsTT8R
35S::RsTT8nR
35S::RsMYB1.1nR
+
35S::RsTT8nR
N. tabacum
35S::GFP
35S::GFP
35S::RsMYB1.1nR
+
35S::RsTT8nR
35S::RsMYB1.1nR
35S::RsMYB1.1R
35S::RsMYB1.1R
+
35S::RsTT8nR
35S::RsTT8nR
35S::RsTT8R
35S::RsMYB1.1nR
+
35S::RsTT8R
35S::RsMYB1.1R
+
35S::RsTT8R
Figure S5. Transient expression of RsMYB1.1 and RsTT8 in radish and tobacco leaves. RsMYB1.1 and RsTT8 of WK10039 (nR) and CUR034 (R) under the control of 35S promoter were expressed individually or coexpressed in WK10039 (upper panel) and N. tabacum (lower panel) leaves. Photographs were obtained 5 days after Agrobacterium infiltration.

## Slide 6
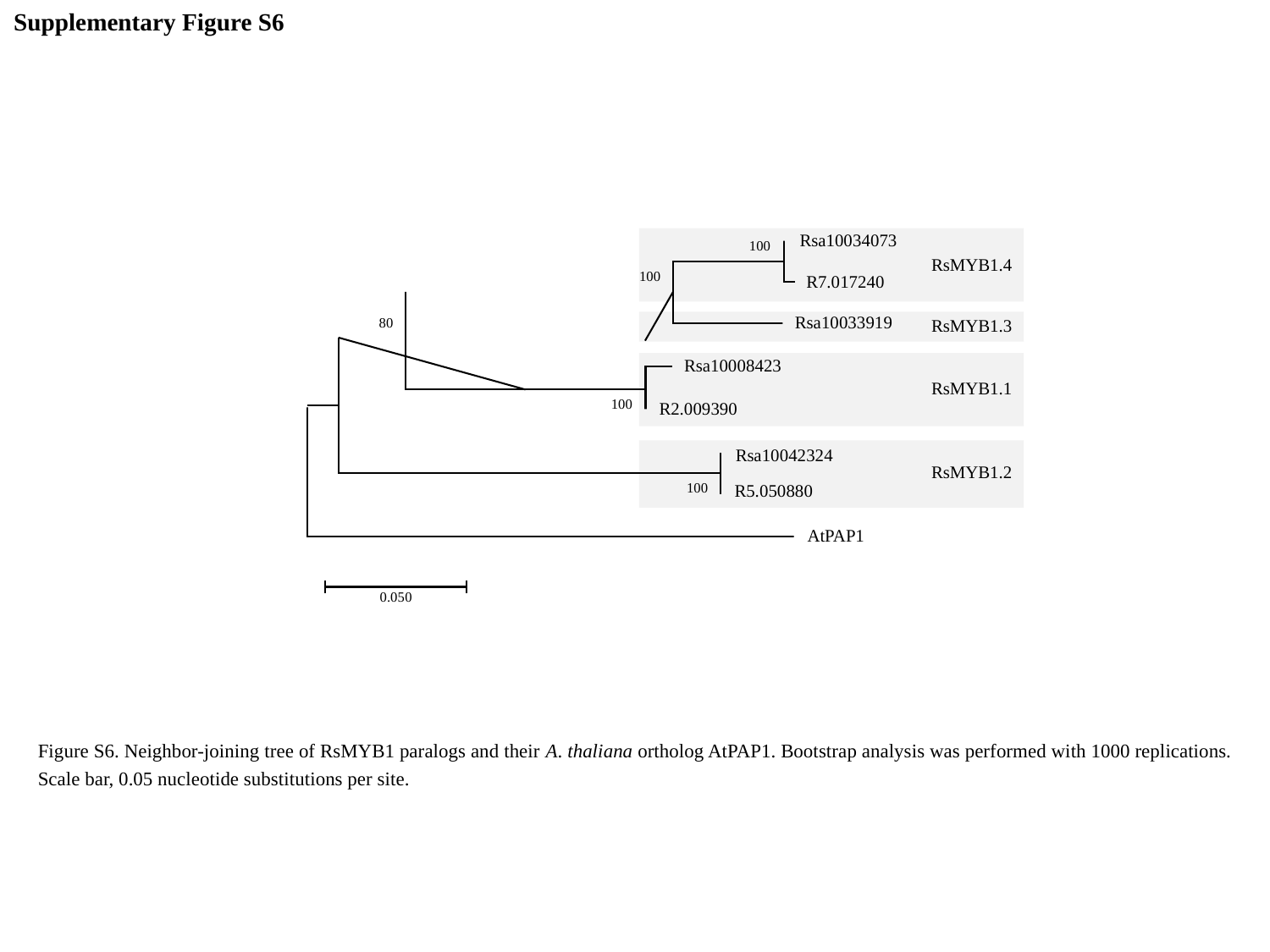

Supplementary Figure S6
Rsa10034073
100
RsMYB1.4
100
R7.017240
Rsa10033919
80
RsMYB1.3
Rsa10008423
RsMYB1.1
100
R2.009390
Rsa10042324
RsMYB1.2
100
R5.050880
AtPAP1
0.050
Figure S6. Neighbor-joining tree of RsMYB1 paralogs and their A. thaliana ortholog AtPAP1. Bootstrap analysis was performed with 1000 replications. Scale bar, 0.05 nucleotide substitutions per site.
