# Supplementary material for: Effect of structural variation in the promoter region of RsMYB1.1 on the skin color of radish taproot
Source: Front Plant Sci. 2024 Jan 8;14:1327009. doi: 10.3389/fpls.2023.1327009 (PMC10804855; doi:10.3389/fpls.2023.1327009)
Supplement: Supplementary file 2 [file Table_1.docx]

Table S1. Primers used in this study.

| Type | Primer name | Forward primer (5’ to 3’) | Reverse primer (5’ to 3’) | Extension primer (5’ to 3’) |
| --- | --- | --- | --- | --- |
| Genotyping | gt-pRsMYB1.1 | GGACCAAGGATAAATACTAGAAGTG | TGGATTTGTTGTTAAACGYTTAGT |  |
|  | gt-RsMYB1.1-E3 | ACGTTGGATGTTCACGGTTAACAACGGCTG | ACGTTGGATGCTTCCACCACATGTTCTCTC |  |
|  | e1 |  |  | AACGTCAACTTTTGGCT |
|  | e2 |  |  | AGTTGACGTTATTCCTCTA |
|  | e3 |  |  | TCCGCATCTTTTTTACATG |
| Cloning | c-pMYB1.1^nR^-full | TGGATTTGTTGTTAAACGYTTAGT | CCCATGGGTATTGATATCATCTTCTGTCAC |  |
|  | c-pMYB1.1^nR^-d1 | CCTGCAGGGAAGTTAAATGGGCGATATGA | CCCATGGGTATTGATATCATCTTCTGTCAC |  |
|  | c-pMYB1.1^nR^-d2 | CCTGCAGTCAACAAAATCATAATACGGGTC | CCCATGGGTATTGATATCATCTTCTGTCAC |  |
|  | c-pMYB1.1^nR^-d3 | TGGATTTGTTGTTAAACGYTTAGT | CCTGCAGGTATTGATATCATCTTCTGTCAC |  |
|  | c-pMYB1.1^nR^-d4 | CAACCTCGGTGTTTGACGTTGAAC | CCCATGGGTATTGATATCATCTTCTGTCAC |  |
|  | c-pMYB1.1^R^-full | TGGATTTGTTGTTAAACGYTTAGT | CCCATGGGTATTGATATCATCTTCTGTCAC |  |
| Y2H | y-RsMYB1.1 | GCGAATTCGAGGGTTCGCCAAAAGGTTT | ATCCCGGGCTAATCAAGTTCAACAGTCTCT |  |
|  | y-RsTT8^nR^ | GCGAATTCGATGAATCAAGTATTATACCGG | ATCCCGGGCTAGGAACTAGAGTTTATTTTGA |  |
|  | y-RsTT8^R^ | GCGAATTCGATGAATCAAGTATTATACCGG | ATCCCGGGCTAGAGTTTATTTTGAGATATGAT |  |
| qPCR | q-RsActin7-2 | CTCGTTGTAGAAAGTGTGATG | AAGATGCTTACGTTGGTGAC |  |
|  | q-RsMYB1.1 | GAAACAATTGCACACTAAATGCTC | CAACCTCGGTGTTTGACGTTGAAC |  |
|  | q-RsTT8 | AAGCCTGCTCTTTTTGAACACTC | TGAGAATAACTTCCGCCTTCCTC |  |
|  | q-RsTTG1 | AAGGTTGTGATTCTCGACATTCG | TCCGATTCTGATCACTTCCCATC |  |
|  | q-RsCHS | TGAGATCAGAAAGGCACAGAGAG | CGCACATGTTAGGGTTCTCTTTC |  |
|  | q-RsDFR | TGTACGAACAAACTGTTGCCAAG | TTCCACCAACATATCCTCGAGAC |  |
|  | q-RsANS | GGACAGCTTGAGTGGGAAGATTA | AAGCCACCAACCTCTTTCTCTAG |  |

Table S2. Phenotypic traits and Illumina read mapping statistics to the Rs2.0 reference genome of radish genotypes.

| Sample ID | Root skin color | Phenotype  group | Total bases (bp) | Total reads | Mapping rate (%) | | | Reference  coverage (%) | Read  depth (×) |
| --- | --- | --- | --- | --- | --- | --- | --- | --- | --- |
|  |  |  |  |  | PE^a^ | SE^b^ | Un^c^ |  |  |
| WK10039 | White | non-Red | 108,627,596,996 | 552,618,445 | 96.0 | 0.0 | 4.0 | 98.2 | 141.13 |
| WK10024 | Red | Red | 65,531,063,518 | 347,845,620 | 96.9 | 0.5 | 2.6 | 91.7 | 132.60 |
| Long_Scarlet | Red | Red | 11,977,854,236 | 61,062,826 | 97.4 | 0.7 | 1.9 | 88.9 | 25.80 |
| DB102 | Red | Red | 10,988,919,396 | 55,920,836 | 97.1 | 0.8 | 2.2 | 86.6 | 24.18 |
| DB104 | White | non-Red | 11,447,666,484 | 58,285,487 | 98.1 | 0.4 | 1.5 | 86.7 | 26.00 |
| DB109 | White | non-Red | 12,307,553,782 | 62,733,879 | 97.8 | 0.4 | 1.8 | 86.7 | 27.71 |
| DB110 | White | non-Red | 11,592,765,566 | 58,864,475 | 98.0 | 0.4 | 1.6 | 87.2 | 26.28 |
| DB113 | White | non-Red | 11,815,731,542 | 60,059,863 | 97.9 | 0.4 | 1.7 | 86.8 | 26.61 |
| Aokubi | White | non-Red | 74,899,371,526 | 384,301,024 | 98.5 | 0.1 | 1.4 | 90.9 | 162.58 |
| Sayatori | White | non-Red | 10,456,700,992 | 52,290,067 | 96.2 | 0.6 | 3.2 | 86.1 | 23.19 |
| Raphanistroides1 | White | non-Red | 11,819,862,608 | 59,605,747 | 97.7 | 0.5 | 1.9 | 88.9 | 26.93 |
| Raphanistroides2 | White | non-Red | 11,708,829,194 | 59,090,742 | 97.6 | 0.5 | 2.0 | 90.1 | 26.08 |
| Raphanistroides3 | White | non-Red | 11,463,324,968 | 57,901,036 | 97.7 | 0.5 | 1.8 | 89.5 | 25.82 |
| Wild_radish1 | White | non-Red | 11,250,880,490 | 57,117,969 | 97.0 | 0.5 | 2.5 | 90.5 | 23.73 |
| Wild_radish2 | White | non-Red | 11,380,020,716 | 57,843,215 | 97.4 | 0.6 | 2.0 | 90.2 | 24.23 |
| Wild_radish3 | White | non-Red | 12,125,367,014 | 61,039,790 | 97.7 | 0.5 | 1.8 | 90.5 | 27.13 |
| Raphanistrum | White | non-Red | 26,349,670,040 | 131,770,811 | 94.7 | 0.8 | 4.5 | 84.4 | 33.28 |
| CUR001 | White-Green | non-Red | 9,329,115,562 | 31,865,610 | 99.2 | 0.2 | 0.6 | 82.5 | 23.95 |
| CUR003 | White-Green | non-Red | 9,366,632,896 | 32,002,731 | 99.4 | 0.2 | 0.4 | 82.6 | 23.97 |
| CUR005 | White | non-Red | 8,967,200,226 | 30,612,535 | 99.2 | 0.3 | 0.6 | 82.7 | 22.81 |
| CUR006 | Red | Red | 9,506,646,720 | 32,476,482 | 98.3 | 0.5 | 1.3 | 83.1 | 21.63 |
| CUR007 | Red | Red | 7,999,072,940 | 27,274,615 | 99.2 | 0.3 | 0.5 | 80.5 | 20.83 |
| CUR008 | White | non-Red | 9,358,118,452 | 31,934,124 | 98.8 | 0.3 | 0.9 | 85.8 | 21.22 |
| CUR009 | Red | Red | 10,528,643,868 | 35,951,272 | 98.5 | 0.5 | 1.1 | 83.6 | 24.05 |
| CUR010 | White-Green | non-Red | 8,540,150,448 | 29,190,165 | 99.5 | 0.2 | 0.3 | 85.1 | 21.64 |
| CUR011 | White | non-Red | 8,384,848,898 | 28,647,944 | 99.5 | 0.2 | 0.3 | 81.8 | 22.04 |
| CUR013 | White-Green | non-Red | 10,294,170,214 | 35,185,939 | 98.4 | 0.4 | 1.2 | 84.1 | 23.36 |
| CUR014 | White-Green | non-Red | 10,224,659,114 | 34,861,347 | 99.5 | 0.2 | 0.3 | 83.7 | 26.12 |
| CUR015 | Black | non-Red | 9,563,747,836 | 32,676,282 | 98.6 | 0.3 | 1.1 | 83.9 | 21.93 |
| CUR016 | White-Green | non-Red | 11,707,662,602 | 39,998,636 | 98.5 | 0.4 | 1.1 | 87.2 | 25.87 |
| CUR018 | Red-Purple | Red | 9,531,436,318 | 32,560,055 | 98.4 | 0.4 | 1.2 | 85.0 | 21.53 |
| CUR019 | Red-Purple | Red | 8,549,646,670 | 29,217,372 | 98.4 | 0.4 | 1.2 | 83.5 | 19.53 |
| CUR020 | White | non-Red | 8,653,085,366 | 29,554,564 | 99.4 | 0.2 | 0.4 | 83.6 | 22.05 |
| CUR021 | White | non-Red | 8,899,737,696 | 30,372,045 | 99.6 | 0.2 | 0.3 | 82.9 | 23.04 |
| CUR022 | Red-Purple | Red | 8,424,050,022 | 28,763,818 | 99.2 | 0.3 | 0.5 | 80.4 | 22.05 |
| CUR023 | White | non-Red | 9,295,779,482 | 31,811,856 | 98.7 | 0.3 | 1.0 | 86.9 | 20.87 |
| CUR024 | Red | Red | 9,871,550,826 | 33,645,152 | 98.6 | 0.5 | 1.0 | 84.5 | 22.29 |
| CUR025 | White | non-Red | 7,899,151,412 | 27,019,880 | 99.2 | 0.3 | 0.6 | 82.1 | 20.44 |
| CUR026 | White-Green | non-Red | 10,534,852,778 | 35,840,947 | 99.5 | 0.2 | 0.4 | 80.5 | 27.66 |
| CUR027 | White-Green | non-Red | 8,950,103,966 | 30,574,276 | 99.5 | 0.1 | 0.4 | 83.6 | 23.06 |
| CUR028 | White-Green | non-Red | 8,884,700,576 | 30,363,308 | 99.0 | 0.3 | 0.7 | 84.3 | 20.67 |
| CUR029 | White-Green | non-Red | 8,508,999,922 | 29,074,624 | 99.4 | 0.2 | 0.4 | 82.5 | 21.98 |
| CUR030 | Red | Red | 7,780,641,470 | 26,625,964 | 99.5 | 0.2 | 0.4 | 82.9 | 20.11 |
| CUR031 | White-Green | non-Red | 9,322,520,448 | 31,697,953 | 99.5 | 0.2 | 0.3 | 84.9 | 23.50 |
| CUR032 | White | non-Red | 11,365,278,022 | 38,873,644 | 98.9 | 0.3 | 0.9 | 85.2 | 26.02 |
| CUR034 | Red | Red | 10,086,998,676 | 34,533,916 | 98.4 | 0.5 | 1.1 | 84.4 | 22.96 |
| CUR037 | White | non-Red | 9,888,021,914 | 33,886,845 | 98.6 | 0.5 | 1.0 | 84.8 | 22.42 |
| CUR038 | White-Green | non-Red | 8,061,640,260 | 27,570,097 | 99.0 | 0.3 | 0.7 | 81.5 | 20.70 |
| CUR039 | White-Green | non-Red | 10,906,479,282 | 37,456,913 | 98.6 | 0.3 | 1.1 | 84.8 | 25.08 |
| CUR040 | Red | Red | 7,867,166,216 | 26,891,412 | 99.4 | 0.2 | 0.4 | 83.5 | 20.26 |
| CUR044 | Red | Red | 9,567,532,334 | 32,884,153 | 98.4 | 0.4 | 1.2 | 83.5 | 22.02 |
| CUR046 | White-Green | non-Red | 8,488,844,754 | 28,822,184 | 99.3 | 0.2 | 0.4 | 83.9 | 21.49 |
| CUR047 | Red-Purple | Red | 9,488,625,578 | 32,615,137 | 98.6 | 0.4 | 1.0 | 84.5 | 21.72 |
| CUR048 | White-Green | non-Red | 8,578,220,784 | 29,157,225 | 99.5 | 0.2 | 0.3 | 83.6 | 22.02 |
| CUR049 | White | non-Red | 7,831,712,044 | 26,832,191 | 99.5 | 0.2 | 0.4 | 84.7 | 19.90 |
| CUR050 | White | non-Red | 9,750,164,068 | 33,290,062 | 99.5 | 0.2 | 0.4 | 84.1 | 24.87 |
| CUR051 | White-Green | non-Red | 9,187,891,610 | 31,216,120 | 99.5 | 0.2 | 0.3 | 83.5 | 23.55 |
| CUR052 | White | non-Red | 8,482,497,472 | 28,810,558 | 99.5 | 0.2 | 0.3 | 82.7 | 22.00 |
| CUR054 | White | non-Red | 9,734,025,446 | 33,467,162 | 98.8 | 0.3 | 0.9 | 86.1 | 22.25 |
| CUR055 | White | non-Red | 8,551,275,310 | 29,367,204 | 98.5 | 0.3 | 1.2 | 84.4 | 19.69 |
| CUR056 | White | non-Red | 10,126,025,478 | 34,770,671 | 98.9 | 0.3 | 0.8 | 85.7 | 23.23 |
| CUR058 | White | non-Red | 9,466,369,266 | 32,167,917 | 99.5 | 0.2 | 0.3 | 82.8 | 24.48 |
| CUR059 | Red-Purple | Red | 9,033,484,088 | 31,001,658 | 98.4 | 0.4 | 1.2 | 83.1 | 20.64 |
| CUR061 | White-Green | non-Red | 8,606,027,044 | 29,248,989 | 99.5 | 0.2 | 0.3 | 83.1 | 22.26 |
| CUR063 | Red-Purple | Red | 8,737,365,796 | 29,712,034 | 99.1 | 0.3 | 0.6 | 82.4 | 22.12 |
| CUR064 | White-Green | non-Red | 8,902,107,854 | 30,247,799 | 99.5 | 0.2 | 0.4 | 83.4 | 22.82 |
| CUR066 | Red | Red | 8,243,999,734 | 28,204,953 | 99.4 | 0.2 | 0.4 | 84.6 | 20.73 |
| CUR068 | White | non-Red | 9,681,978,674 | 32,879,134 | 99.5 | 0.2 | 0.4 | 82.5 | 25.11 |
| CUR069 | Black | non-Red | 8,598,458,924 | 29,397,506 | 99.1 | 0.3 | 0.6 | 82.9 | 21.78 |
| CUR071 | Red | Red | 7,760,443,940 | 26,507,350 | 99.5 | 0.2 | 0.4 | 82.3 | 20.24 |
| CUR072 | Red | Red | 8,089,618,548 | 27,568,191 | 99.2 | 0.3 | 0.5 | 80.2 | 21.33 |
| CUR074 | White-Green | non-Red | 7,311,609,194 | 25,003,865 | 98.8 | 0.3 | 0.8 | 81.1 | 18.85 |
| CUR075 | Red-Purple | Red | 8,526,398,106 | 29,236,926 | 98.3 | 0.5 | 1.2 | 82.8 | 19.51 |
| CUR076 | Red-Purple | Red | 10,247,994,236 | 35,091,409 | 98.5 | 0.4 | 1.1 | 83.7 | 23.44 |
| CUR078 | Black | non-Red | 8,869,095,340 | 30,315,837 | 98.3 | 0.5 | 1.2 | 82.5 | 20.52 |
| CUR079 | Red | Red | 8,580,702,994 | 29,286,692 | 98.9 | 0.3 | 0.8 | 82.1 | 21.78 |
| CUR080 | Red | Red | 8,768,858,204 | 30,002,823 | 98.2 | 0.5 | 1.3 | 82.1 | 20.40 |
| CUR081 | Red | Red | 7,579,956,172 | 25,868,072 | 99.1 | 0.3 | 0.6 | 81.6 | 19.45 |
| CUR082 | Red | Red | 8,112,054,568 | 27,655,135 | 99.2 | 0.3 | 0.5 | 80.0 | 21.38 |
| CUR083 | Red | Red | 10,454,605,536 | 35,776,483 | 98.7 | 0.4 | 1.0 | 84.1 | 24.19 |
| CUR084 | Red | Red | 7,740,646,868 | 26,434,852 | 99.1 | 0.3 | 0.6 | 81.5 | 19.92 |
| CUR085 | Red | Red | 9,884,207,622 | 33,787,096 | 98.4 | 0.5 | 1.1 | 83.6 | 22.72 |
| CUR086 | Red | Red | 8,310,517,306 | 28,362,162 | 98.9 | 0.3 | 0.8 | 83.3 | 20.85 |
| CUR087 | Red | Red | 8,011,122,346 | 27,343,442 | 99.4 | 0.2 | 0.4 | 84.1 | 20.33 |
| CUR088 | Red | Red | 7,894,547,816 | 26,914,476 | 99.4 | 0.2 | 0.4 | 81.7 | 20.73 |
| CUR089 | Red | Red | 8,428,472,032 | 28,738,957 | 99.5 | 0.2 | 0.3 | 82.0 | 22.14 |
| CUR091 | Red-Purple | Red | 8,666,265,458 | 29,548,278 | 99.4 | 0.2 | 0.4 | 84.0 | 21.95 |
| CUR094 | White | Red | 9,350,703,928 | 31,885,170 | 99.3 | 0.3 | 0.5 | 82.8 | 23.88 |
| CUR096 | Black | non-Red | 8,497,562,154 | 29,057,117 | 99.2 | 0.3 | 0.5 | 81.8 | 21.71 |
| CUR097 | White-Green | non-Red | 8,374,282,912 | 28,588,324 | 99.4 | 0.2 | 0.4 | 81.0 | 22.06 |
| CUR099 | White | non-Red | 9,183,095,658 | 31,441,158 | 97.1 | 0.5 | 2.4 | 81.5 | 21.05 |
| CUR103 | White | non-Red | 8,352,897,488 | 28,522,216 | 98.3 | 0.4 | 1.3 | 80.7 | 21.08 |
| CUR105 | White | non-Red | 8,149,539,574 | 27,841,572 | 97.5 | 0.5 | 2.0 | 74.7 | 21.37 |
| CUR109 | White | non-Red | 8,981,691,476 | 30,614,299 | 98.8 | 0.4 | 0.8 | 73.6 | 24.71 |
| CUR111 | White | non-Red | 8,995,779,764 | 30,781,045 | 97.9 | 0.6 | 1.6 | 79.8 | 21.10 |
| CUR117 | White | non-Red | 8,050,436,376 | 27,482,636 | 98.7 | 0.5 | 0.9 | 77.9 | 20.88 |
| CUR121 | White | non-Red | 8,597,248,902 | 29,315,577 | 99.5 | 0.2 | 0.3 | 83.3 | 22.15 |
| CUR122 | White | non-Red | 8,884,198,688 | 30,351,493 | 97.9 | 0.6 | 1.5 | 79.9 | 20.72 |
| CUR124 | White | non-Red | 8,892,332,388 | 30,357,895 | 99.4 | 0.2 | 0.4 | 85.7 | 22.19 |
| CUR128 | White | non-Red | 8,729,354,050 | 29,811,805 | 99.4 | 0.2 | 0.4 | 83.6 | 22.11 |

^a^ PE, paired-end reads; ^b^ SE, single-end reads; ^c^ Un, unmapped read.

Table S3. Summary of the 14 SNPs and 21 InDels on the R2 chromosome that reached the suggestive threshold in the GWAS.

| Position (bp) | Type | Reference  base | Alternate  base | –log_10_(*P*)^a^ | Genomic  region | Gene ID | Annotation |
| --- | --- | --- | --- | --- | --- | --- | --- |
| 7,958,481 | InDel | GA | G | 7.54 | Intergenic |  |  |
| 7,959,159 | InDel | GA | G | 5.76 | Intergenic |  |  |
| 7,960,013 | InDel | TTTTTAGGC | T | 6.71 | Intergenic |  |  |
| 7,960,335 | InDel | A | AT | 5.59 | Intergenic |  |  |
| 7,964,349 | InDel | C | CCAAAA | 5.96 | Intergenic |  |  |
| 7,966,739 | InDel | AAT | A | 5.61 | Intron 1 | R2.009320 | Methyltransferase |
| 7,966,029 | SNP | T | C | 7.76 | Exon 2 | R2.009320 |  |
| 7,971,973 | InDel | A | AATCTTGTTGTATTA  TGTAAAGCT | 5.59 | Intergenic |  |  |
| 7,976,342 | InDel | TA | T | 5.96 | Intergenic |  |  |
| 7,985,186 | InDel | TA | T | 6.33 | Intergenic |  |  |
| 7,985,422 | InDel | A | AT | 7.60 | Intergenic |  |  |
| 7,986,414 | InDel | AT | A | 5.98 | Intergenic |  |  |
| 7,986,565 | InDel | CTACAA | C | 5.73 | Intergenic |  |  |
| 7,986,784 | InDel | ATTGAATAAACATAC  TTTTTTTTTGTAACT | A | 5.92 | Intergenic |  |  |
| 7,992,815 | InDel | GAAGGTCTCATATTC  TGCCATGTGTAACGAGC | G | 6.63 | Intergenic |  |  |
| 7,993,391 | SNP | A | G | 7.76 | Intergenic |  |  |
| 7,993,440 | InDel | A | ATGAC | 6.81 | Intergenic |  |  |
| 7,994,055 | InDel | A | AG | 7.24 | Intergenic |  |  |
| 7,998,343 | SNP | C | G | 7.43 | Intron 2 | R2.009350 | bHLH83 |
| 7,998,671 | InDel | TA | T | 6.01 | Intron 2 | R2.009350 |  |
| 7,998,783 | SNP | T | C | 7.56 | Exon 2 | R2.009350 |  |
| 7,999,726 | InDel | A | ATTAG | 7.53 | Intergenic |  |  |
| 8,002,370 | InDel | A | AT | 5.68 | Intergenic |  |  |
| 8,005,103 | InDel | C | CAT | 8.12 | Intergenic |  |  |
| 8,011,705 | SNP | A | T | 7.50 | Intergenic |  |  |
| 8,034,806 | SNP | C | T | 7.44 | Exon 3 | R2.009390 | Myb1.1 |
| 8,036,440 | SNP | A | G | 8.80 | Promoter | R2.009390 |  |
| 8,055,076 | SNP | C | T | 7.43 | Exon 1 | R2.009400 | DELLA protein RGL1 |
| 8,064,830 | InDel | CCCA | C | 5.92 | Intergenic |  |  |
| 8,074,845 | SNP | G | C | 7.88 | Exon 1 | R2.009420 | Pentatricopeptide repeat-containing protein |
| 8,074,862 | SNP | G | A | 7.95 | Exon 1 | R2.009420 |  |
| 8,075,254 | SNP | A | T | 6.33 | Exon 1 | R2.009420 |  |
| 8,075,354 | SNP | G | A | 7.23 | Exon 1 | R2.009420 |  |
| 8,075,363 | SNP | C | T | 7.19 | Exon 1 | R2.009420 |  |
| 8,075,534 | SNP | C | G | 6.00 | Exon 1 | R2.009420 |  |

^a^Suggestive SNP threshold is *P*= 1.26 × 10^-6^ (1/794,484) and suggestive InDel threshold is *P*= 3.24 × 10^-6^ (1/308,433).

Table S4. Summary of the InDels identified near R2.009390.

| Chr | Position (bp) | Reference | Alternate | −log_10_(*P*) | Genomic |
| --- | --- | --- | --- | --- | --- |
|  |  | base | base |  | region^a^ |
| R2 | 8,035,404 | T | TAATG | 4.78 | Intron 1 |
| R2 | 8,035,536 | T | TCTTGCAGG | 1.47 | Intron 1 |
| R2 | 8,035,767 | GT | G | 0.66 | Intron 1 |
| R2 | 8,036,095 | T | TTATATATATATATATATATATATATATATATATATATA | 4.09 | Intron 1 |
| R2 | 8,036,182 | C | CA | 0.40 | Intron 1 |
| R2 | 8,036,829 | T | TA | 1.20 | promoter |
| R2 | 8,037,423 | T | TTA | 0.12 | promoter |
| R2 | 8,037,790 | AT | A | 0.62 | promoter |
| R2 | 8,037,996 | CT | C | 1.24 | promoter |
| R2 | 8,038,528 | A | AACCAATGT | 0.88 | promoter |
| R2 | 8,038,659 | A | ATATAAATATAACTTCT | 1.24 | promoter |
| R2 | 8,039,498 | AT | A | 1.18 | promoter |
| R2 | 8,040,092 | C | CTTT | 3.41 | promoter |
| R2 | 8,040,092 | C | CT | 0.48 | promoter |
| R2 | 8,040,727 | C | CA | 4.21 | promoter |
| R2 | 8,041,028 | TAAA | T | 0.40 | promoter |
| R2 | 8,041,124 | T | TG | 1.64 | promoter |
| R2 | 8,041,299 | T | TC | 0.11 | promoter |
| R2 | 8,041,301 | C | CG | 2.48 | promoter |
| R2 | 8,041,427 | AT | A | 0.96 | promoter |
| R2 | 8,041,431 | AAAAAACTTGTTATGTGGTTGTGACAATGTTAGAAATTATTTT | A | 0.45 | promoter |
| R2 | 8,041,536 | A | AAC | 3.53 | promoter |
| R2 | 8,041,732 | T | TA | 0.74 | promoter |
| R2 | 8,041,855 | CT | C | 2.58 | promoter |
| R2 | 8,042,314 | CTAAA | C | 0.99 | Intergenic |
| R2 | 8,042,744 | TA | T | 3.44 | Intergenic |
| R2 | 8,042,970 | GT | G | 0.62 | Intergenic |
| R2 | 8,043,674 | G | GT | 3.47 | Intergenic |
| R2 | 8,043,914 | A | AAT | 0.46 | Intergenic |
| R2 | 8,044,031 | C | CT | 0.54 | Intergenic |
| R2 | 8,044,140 | G | GA | 0.43 | Intergenic |
| R2 | 8,044,364 | C | CT | 0.99 | Intergenic |
| R2 | 8,047,914 | C | CT | 1.15 | Intergenic |
| R2 | 8,049,171 | C | CA | 0.18 | Intergenic |
| R2 | 8,049,814 | AT | A | 0.54 | Intergenic |
| R2 | 8,050,309 | T | TTATA | 0.01 | Intergenic |
| R2 | 8,050,524 | AT | A | 2.60 | Intergenic |
| R2 | 8,050,683 | CA | C | 0.83 | Intergenic |
| R2 | 8,051,656 | G | GA | 0.41 | Intergenic |
| R2 | 8,052,005 | GA | G | 0.15 | Intergenic |
| R2 | 8,052,481 | C | CAACAACTCTTT | 1.71 | Intergenic |
| R2 | 8,053,061 | AT | A | 2.26 | Intergenic |
| R2 | 8,053,297 | T | TA | 0.70 | Intergenic |

^a^ Promoter region was defined as the 5 kb upstream sequence from the start codon.

Table S5. Copy numbers of the RsIS in the genomes of radishes, diploid *Brassica*, and *Arabidopsis thaliana*.

| Species | Accession | Copy number |
| --- | --- | --- |
| *R. sativu*s cv. WK10039 | JRUI00000000.3 | 144 |
| *R. sativus* var. *longipinatus* (Rs00) | GWHANWD00000000 | 158 |
| *R. sativus* var. *caudatu*s (Rs01) | GWHANWE00000000 | 183 |
| *R. sativus* var. *oleiformis* (Rs02) | GWHANWJ00000000 | 158 |
| *R. sativus* var. *niger* (Rs03) | GWHANWK00000000 | 132 |
| *R. sativus* var. *longipinatus* (Rs04) | GWHANWR00000000 | 149 |
| *R. sativus* var. *longipinatus* (Rs05) | GWHANWQ00000000 | 156 |
| *R. sativus* var. *radicula* (Rs06) | GWHANWP00000000 | 139 |
| *R. sativus* var*. raphanistroides* (Rs07) | GWHANWS00000000 | 138 |
| *R. raphanistrum* ssp. *landra* (Rs08) | GWHANWL00000000 | 153 |
| *R. raphanistrum* ssp. *raphanistrum* (Rs09) | GWHANWM00000000 | 138 |
| *R. raphanistrum* × *R. sativus* (Rs10) | GWHANWN00000000 | 137 |
| *B. rapa* | GWHAAES00000000 | 0 |
| *B. nigra* | PRJNA516907 | 0 |
| *B. oleracea* | GCF_000695525.1 | 0 |
| *A. thaliana* | TAIR10 | 0 |

Table S6. Distribution of the RsIS in the *R. sativus* cv. WK10039 genome.

| Chr. | Promoter | Intron | Intergenic region | Total |
| --- | --- | --- | --- | --- |
| R1 | 3 | 2 | 8 | 13 |
| R2 | 10 | 3 | 16 | 29 |
| R3 | 2 | 1 | 3 | 6 |
| R4 | 2 | 2 | 16 | 20 |
| R5 | 5 | 3 | 8 | 16 |
| R6 | 7 | 1 | 18 | 26 |
| R7 | 3 | 1 | 6 | 10 |
| R8 | 5 | 2 | 7 | 14 |
| R9 | 6 | 0 | 4 | 10 |
| Total | 43 | 15 | 86 | 144 |

Table S7. Average TMM values determined by RNA-seq using three biological replicates of 43 genes with RsIS in their promoter regions.

| Gene ID | Description | Leaf  (2 weeks) | Leaf  (8 weeks) | Root  (2 weeks) | Root  (8 weeks) | Anther | Petal | Carpel | Average |
| --- | --- | --- | --- | --- | --- | --- | --- | --- | --- |
| R1.022030 | Protein indeterminate-domain 16 | 1.2 | 1.1 | 1.0 | 0.1 | 3.3 | 2.0 | 7.8 | 2.4 |
| R1.030390 | Hypothetical protein | 0.1 | 0.2 | 0.3 | 0.1 | 2.7 | 0.5 | 1.7 | 0.8 |
| R1.034080 | Histone H2A variant 3 | 35.3 | 55.0 | 25.9 | 352.9 | 54.2 | 72.2 | 62.0 | 93.9 |
| R2.009390 | RsMYB1.1 | 2.1 | 4.6 | 0.1 | 0.3 | 0.4 | 0.7 | 0.1 | 1.2 |
| R2.014990 | Uncharacterized protein | 0.1 | 0.3 | 0.4 | 0.6 | 0.8 | 0.6 | 0.8 | 0.5 |
| R2.015360 | Glycosyltransferase family protein 64 protein C5 | 0.0 | 0.0 | 0.0 | 0.0 | 6.0 | 0.0 | 0.0 | 0.9 |
| R2.016940 | Protein DA1-related 4 | 0.7 | 1.2 | 0.1 | 0.1 | 0.2 | 0.0 | 0.0 | 0.3 |
| R2.032340 | PPM-type phosphatase domain protein | 0.0 | 0.0 | 0.0 | 0.0 | 0.0 | 0.0 | 0.0 | 0.0 |
| R2.035860 | ADP-ribosylation factor GTPase-activating protein AGD8 | 19.7 | 40.1 | 24.9 | 51.3 | 30.0 | 29.1 | 44.5 | 34.2 |
| R2.038670 | F-box protein | 0.0 | 0.0 | 0.0 | 0.0 | 0.0 | 0.0 | 0.0 | 0.0 |
| R2.039800 | SPX domain-containing membrane protein | 25.0 | 14.7 | 1.7 | 2.2 | 26.6 | 12.3 | 0.4 | 11.8 |
| R2.045040 | Mannan endo-1,4-beta-mannosidase 5 | 18.2 | 32.2 | 16.9 | 19.9 | 4.4 | 6.3 | 9.3 | 15.3 |
| R2.054350 | Polycomb group protein EMBRYONIC FLOWER 2 | 27.1 | 16.4 | 18.6 | 11.5 | 40.8 | 36.4 | 41.3 | 27.5 |
| R3.005110 | Uncharacterized protein | 0.1 | 0.0 | 0.1 | 0.6 | 0.8 | 0.6 | 0.1 | 0.3 |
| R3.007090 | Leucine-rich repeat protein kinase family protein | 43.3 | 32.4 | 9.1 | 13.2 | 13.2 | 17.8 | 14.5 | 20.5 |
| R4.042540 | FBD-associated F-box protein | 4.7 | 14.8 | 5.5 | 22.1 | 5.9 | 15.2 | 16.1 | 12.0 |
| R4.057780 | Hypothetical protein | 0.0 | 0.0 | 0.0 | 0.0 | 0.0 | 0.0 | 0.0 | 0.0 |
| R5.013310 | Winged-helix DNA-binding transcription factor family protein | 1.0 | 26.0 | 1.6 | 0.6 | 1.6 | 8.1 | 2.0 | 5.8 |
| R5.027550 | Beta-hexosaminidase 2 | 30.5 | 23.4 | 7.1 | 0.8 | 20.5 | 29.7 | 70.7 | 26.1 |
| R5.034820 | DUF4283 domain-containing protein | 0.0 | 0.0 | 0.0 | 0.0 | 0.0 | 0.0 | 0.0 | 0.0 |
| R5.036410 | Agenet domain, plant type protein | 0.0 | 0.0 | 0.0 | 0.0 | 0.0 | 0.0 | 0.0 | 0.0 |
| R5.037800 | Callose synthase 3 | 0.0 | 0.0 | 0.0 | 0.0 | 0.0 | 0.0 | 0.0 | 0.0 |
| R6.030160 | Uncharacterized protein | 1.6 | 1.8 | 1.2 | 1.2 | 2.3 | 3.8 | 3.6 | 2.2 |
| R6.032620 | Mitochondrial outer membrane import complex protein METAXIN | 14.2 | 22.9 | 16.3 | 19.7 | 21.9 | 20.6 | 22.1 | 19.7 |
| R6.042620 | Cyclin-SDS | 1.0 | 0.0 | 0.3 | 1.1 | 0.9 | 0.5 | 1.2 | 0.7 |
| R6.051940 | Auxin transporter-like protein 2 | 9.6 | 6.6 | 20.5 | 2.1 | 4.5 | 27.0 | 9.5 | 11.4 |
| R6.053710 | GDP-mannose 4,6 dehydratase 1 | 0.2 | 0.1 | 0.1 | 0.0 | 0.0 | 0.2 | 0.2 | 0.1 |
| R6.054680 | Cytokinin riboside 5'-monophosphate phosphoribohydrolase LOG1 | 58.8 | 43.7 | 6.6 | 11.9 | 24.4 | 9.6 | 0.9 | 22.3 |
| R6.065090 | Transcription factor bHLH129 | 7.8 | 12.8 | 25.6 | 44.6 | 2.0 | 1.2 | 0.1 | 13.4 |
| R7.025640 | LRR receptor-like serine/threonine-protein kinase | 10.9 | 11.5 | 8.3 | 53.3 | 5.3 | 2.7 | 4.5 | 13.8 |
| R7.031480 | Core-2/I-branching beta-1,6-N-acetylglucosaminyltransferase family protein | 11.7 | 15.2 | 17.6 | 10.8 | 19.2 | 23.3 | 20.3 | 16.9 |
| R7.034150 | Uncharacterized protein | 0.0 | 0.0 | 0.0 | 0.0 | 0.3 | 0.0 | 0.0 | 0.0 |
| R8.002660 | Uncharacterized protein | 0.4 | 1.5 | 1.9 | 2.1 | 0.7 | 2.3 | 2.1 | 1.6 |
| R8.015400 | ADP-ribosyl cyclase/cyclic ADP-ribose hydrolase | 0.0 | 0.0 | 0.0 | 0.0 | 0.0 | 0.0 | 0.0 | 0.0 |
| R8.022990 | AT-hook motif nuclear-localized protein 13 | 22.2 | 32.2 | 32.4 | 65.6 | 58.5 | 32.7 | 37.6 | 40.2 |
| R8.025480 | Hypothetical protein | 0.0 | 0.0 | 0.0 | 0.0 | 0.0 | 0.0 | 0.0 | 0.0 |
| R8.027480 | Uncharacterized protein | 0.2 | 0.3 | 0.1 | 0.0 | 0.0 | 0.1 | 0.2 | 0.1 |
| R9.007990 | Transcription factor BEE 3 | 8.0 | 8.4 | 6.1 | 3.2 | 3.7 | 14.7 | 0.0 | 6.3 |
| R9.018910 | Molybdenum cofactor sulfurase family protein | 54.2 | 43.7 | 17.0 | 54.2 | 43.2 | 38.1 | 39.5 | 41.4 |
| R9.025460 | RING/U-box superfamily protein | 21.6 | 33.8 | 27.5 | 42.3 | 58.7 | 34.4 | 33.2 | 35.9 |
| R9.025800 | Zinc finger CCCH domain-containing protein 19 | 62.1 | 42.1 | 70.5 | 4.6 | 75.6 | 102.8 | 80.9 | 62.6 |
| R9.029100 | Plant invertase/pectin methylesterase inhibitor superfamily protein | 0.0 | 0.0 | 0.0 | 0.0 | 0.0 | 0.0 | 0.0 | 0.0 |
| R9.044030 | Uncharacterized protein | 0.0 | 0.0 | 0.0 | 0.0 | 0.0 | 0.0 | 0.0 | 0.0 |
